# Structural mechanism for the selective phosphorylation of DNA-loaded MCM double hexamers by the Dbf4-dependent kinase

**DOI:** 10.1038/s41594-021-00698-z

**Published:** 2021-12-28

**Authors:** Julia F. Greiwe, Thomas C. R. Miller, Julia Locke, Fabrizio Martino, Steven Howell, Anne Schreiber, Andrea Nans, John F. X. Diffley, Alessandro Costa

**Affiliations:** 1 Macromolecular Machines Laboratory, The Francis Crick Institute, London, UK; 2 Center for Chromosome Stability, University of Copenhagen, Copenhagen, Denmark; 3 Proteomics Science Technology Platform, The Francis Crick Institute, London, UK; 4 Cellular Degradation Systems Laboratory, The Francis Crick Institute, London, UK; 5 Structural Biology Science Technology Platform, The Francis Crick Institute, London, UK; 6 Chromosome Replication Laboratory, The Francis Crick Institute, London, UK; 7 Present Address: Human Technopole, Milan, Italy

**Keywords:** Cryoelectron microscopy, Kinases, Origin firing

## Abstract

Loading of the eukaryotic replicative helicase onto replication origins involves two MCM hexamers forming a double hexamer (DH) around duplex DNA. During S phase, helicase activation requires MCM phosphorylation by Dbf4-dependent kinase (DDK), comprising Cdc7 and Dbf4. DDK selectively phosphorylates loaded DHs, but how such fidelity is achieved is unknown. Here, we determine the cryogenic electron microscopy structure of *Saccharomyces cerevisiae* DDK in the act of phosphorylating a DH. DDK docks onto one MCM ring and phosphorylates the opposed ring. Truncation of the Dbf4 docking domain abrogates DH phosphorylation, yet Cdc7 kinase activity is unaffected. Late origin firing is blocked in response to DNA damage via Dbf4 phosphorylation by the Rad53 checkpoint kinase. DDK phosphorylation by Rad53 impairs DH phosphorylation by blockage of DDK binding to DHs, and also interferes with the Cdc7 active site. Our results explain the structural basis and regulation of the selective phosphorylation of DNA-loaded MCM DHs, which supports bidirectional replication.

## Abstract (web summary)

A cryo-EM structure of the Dbf4-dependent kinase (DDK) in the act of phosphorylating an Mcm2-7 helicase complex reveals how DDK selectively targets DNA-loaded MCM double hexamers to trigger replication origin activation. Checkpoint kinase Rad53 blocks origin firing by impairing double hexamer engagement by DDK.

## Main

DNA replication is tightly regulated to ensure that one accurate copy of each chromosome is inherited by two daughter cells^1^. Replication start sites (origins) are activated in temporally separated steps^2–5^, which are under the control of three classes of protein kinase. These include DDK and cyclin-dependent kinase (CDK) required for replication fork establishment, and S phase checkpoint kinases that block late origin firing if DNA damage is detected^6–11^. During G1 phase, two copies of the hexameric MCM motor of the replicative helicase are loaded onto origins via a concerted and sequential mechanism^12–14^. In this multistep process, a first MCM–Cdt1 assembly is recruited onto DNA by the origin recognition complex (ORC) and the helicase loading factor Cdc6. This MCM motor is then locked around DNA, creating a new site for ORC interaction, which in turn promotes the recruitment of a second MCM motor. The end result of the loading reaction, which depends on ATP hydrolysis by MCM, is the formation of a DH ring around duplex DNA^15–23^. This is a catalytically inactive form of the helicase, where the two MCM rings are arranged in a symmetric configuration, ready to trigger bidirectional origin DNA opening following cell-cycle progression into S phase. Origin activation depends upon DDK, which phosphorylates loaded DHs, rendering them competent for the recruitment of a set of firing factors^7,24–26^. Amongst these factors, Sld2 and Sld3 are phosphorylated by CDK and in turn recruit the helicase activators GINS and Cdc45 (refs. ^6,9,10,27^). Formation of two Cdc45-MCM-GINS (CMG) helicases promotes DH separation and origin DNA untwisting, while recruitment of the Mcm10 firing factor triggers bidirectional replication fork establishment^2,28^. A further level of control occurs in S phase, when checkpoint kinase Rad53 responds to DNA damage detection by blocking late origin firing through inhibition of the CDK and DDK pathways. To achieve this, Rad53 phosphorylates Dbf4 and Sld3, in turn preventing phosphorylation of DHs and recruitment of Cdc45 on the path to CMG formation^8,11^.

Describing the molecular interactions between DDK and MCM is important to understand how origin firing is regulated^7^. Several studies provide insights into the MCM phosphorylation mechanism by DDK. Functional characterization established that DDK docks onto the Mcm2 subunit and phosphorylates the Mcm6 and Mcm4 subunits of the helicase ring, although only Mcm4 modification is essential for origin activation^24,26,29–32^. DDK is a heterodimeric kinase formed by the Cdc7 catalytic subunit, which is wrapped by the Dbf4 activator^33^, containing three structured domains. One is the N-terminal domain, containing a BRCT motif^34^, which has been implicated in DDK docking to DHs^24,29^. The second module is formed by the middle (M) domain, which is intertwined with the Cdc7 kinase insert 2 (KI2) activation loop element. This insert pins the Cdc7 activation loop to the M domain^35^, suggesting that Dbf4-mediated protein interaction might modulate kinase function. The third module is a universally conserved C (C-terminal) zinc finger domain essential for MCM phosphorylation and cell-cycle progression^33,36^.

It is established that Rad53 blocks DH phosphorylation by Cdc7, in turn by phosphorylating Dbf4 (refs. ^8,11^), but the molecular mechanism is unclear. It is in fact unknown whether Dbf4 phosphorylation by Rad53 impairs the interaction between DDK and the DH, or rather the phosphorylated DDK productively engages the DH but fails to catalyze the phosphorylation reaction. To complicate matters, a recent study uncovered a structural function for Rad53 that antagonistically binds DDK, independent of Dbf4 phosphorylation, and contributes to impairment of the physical interaction between DDK and DHs^29^.

Despite the large body of knowledge on origin licensing and activation, several fundamental questions remain to be addressed. For example, while it is clear that ATP binding and hydrolysis by MCM are essential for MCM loading onto origin DNA, which ATPase sites are required and in what step during the DH loading reaction remain unresolved issues. Likewise, it remains to be established how DDK can selectively catalyze activating phosphorylation of loaded DHs that are poised to start bidirectional replication. Finally, how Rad53 interferes with DH phosphorylation by DDK to block late origin firing is unclear.

To address these questions, we imaged DH phosphorylation by DDK using cryogenic electron microscopy (cryo-EM). Our results, complemented by mass spectrometry and biochemical analysis, explain the mechanism and regulation of a key step towards the activation of eukaryotic origins of replication.

## Results

### 3.0-Å-resolution structure of DNA-bound MCM DH

Once loaded onto origins, although DHs do not engage in ATPase-driven translocation they can slide passively along the double helix and off the ends of a linear DNA substrate^3,5,15,37^. For this reason, solving a structure of the DH bound to a linear DNA substrate remains challenging. Previous efforts by us and also the Stillman, Speck and Li laboratories led to cryo-EM structures of limited resolution^15,20^. More recently we observed that loaded DHs can be retained on DNA segments as short as one single replication origin sequence (151 base pairs (bp)), when DNA is capped at both ends with a nucleosome or a covalent HpaII methyltransferase (MH) roadblock. In the same study, we also identified conditions for cryo-EM imaging of the full MCM loading process in solution, and the identification of previously unknown reaction intermediates led us to describe the helicase loading mechanism^13^.

**Fig. 1:**
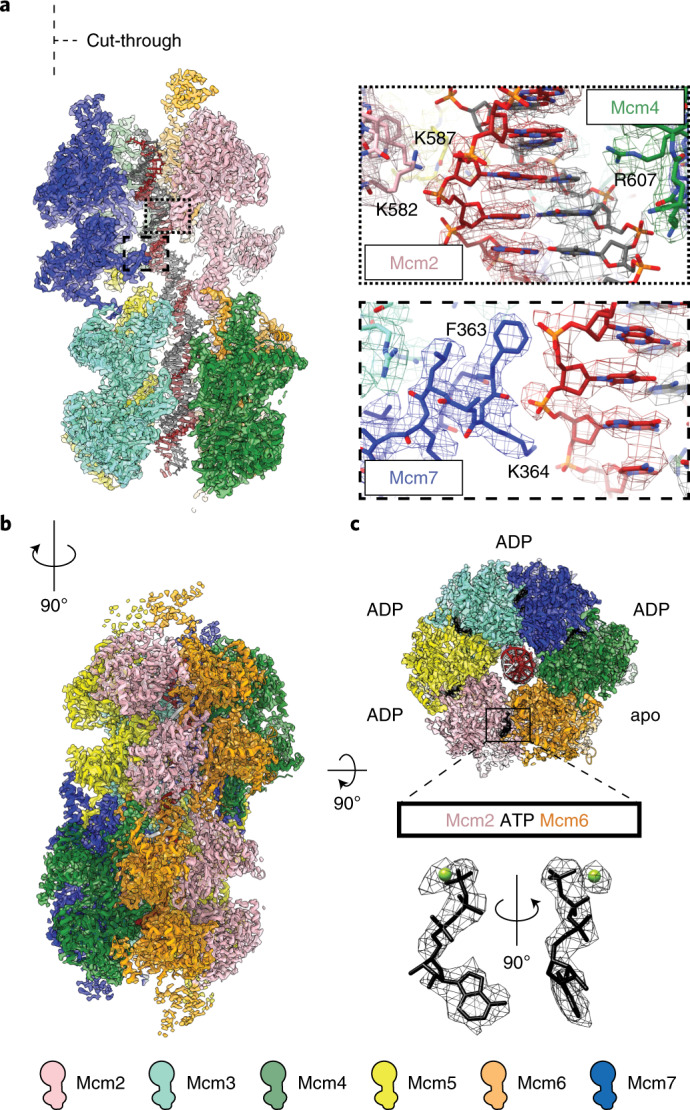
Protein–DNA contacts and nucleotide occupancy in the DNA-loaded MCM DH. **a**, A cut-through view of the MCM DH revealing the atomic model of the double helix. Density and atomic model for selected DNA interacting residues. **b**, Isosurface representation of the 3.0-Å-resolution structure of the DNA-loaded MCM DH. **c**, Bottom view of ATPase sites with nucleotides are depicted in black. Nucleotide density at the Mcm6–Mcm2 interface is consistent with ATP rather than ADP binding. Segmented density for ATP and the magnesium ion in the active site provides confidence about the assignment. Apo means no nucleotide.

**Extended Data Fig. 1:**
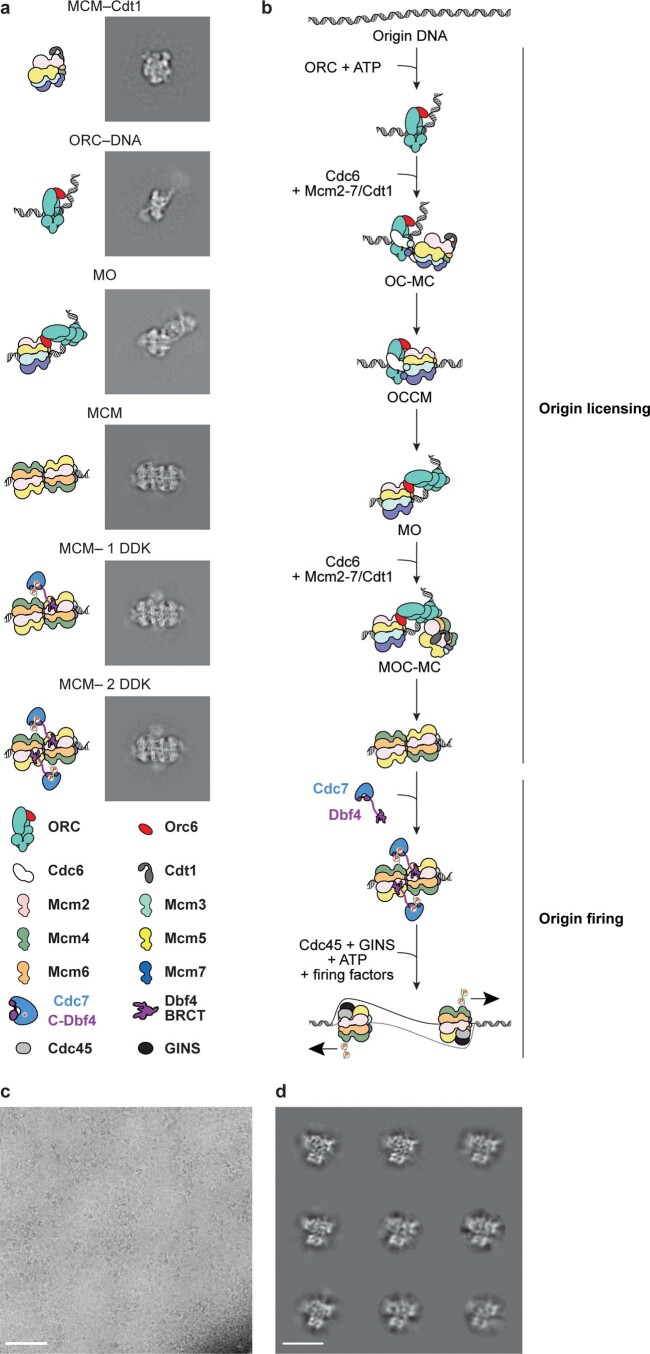
MCM loading and phosphorylation intermediates visualised by cryo-EM. **a** MCM double hexamer loading and phosphorylation was reconstituted *in vitro* and analysed by cryo-EM. As a result, several intermediates on route to double hexamer loading could be visualised, alongside instances where DDK interacts with double hexamers. These intermediates include DNA-bound ORC, MCM–Cdt1 and the MCM–ORC (MO) intermediate. **b** A cartoon representation of the origin licensing. **c** Representative aligned movie sum of yeast DDK analysed by cryo-EM (9,469 movies were collected). Scale bar is equivalent to 50 nm. **d** 2D averages of DDK showing high-resolution features. Scale bar is equivalent to 10 nm.

**Extended Data Fig. 2:**
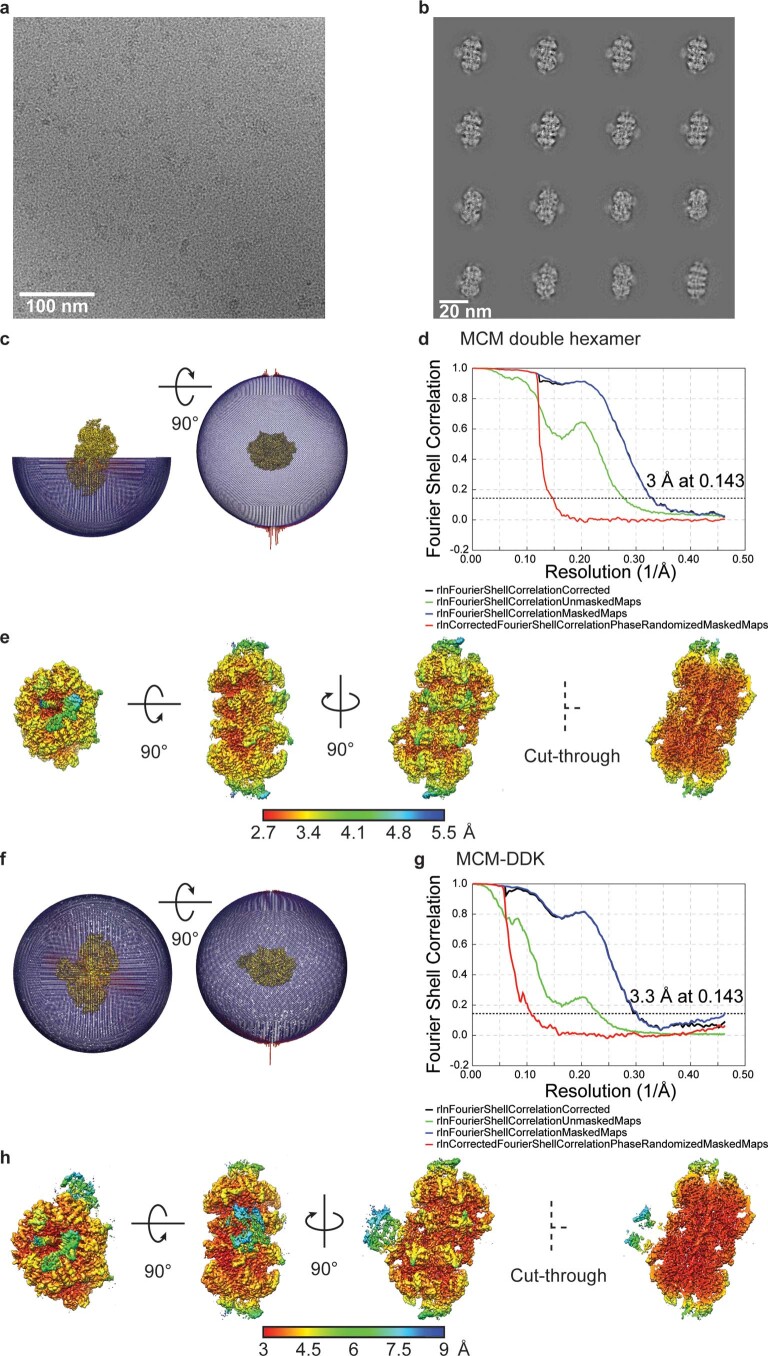
Cryo-EM structure of the MCM double hexamer isolated and bound to DDK. **a** Representative aligned movie sum. **b** 2D averages containing recognisable double hexamers. **c** Angular distribution of the MCM double hexamer structure. **d** Resolution of the MCM double hexamer estimated using gold-standard Fourier Shell Correlation. **e** Three rotated and one cut-through view of the MCM double hexamer 3D structure, color-coded according to the local resolution. **f** Angular distribution of the MCM double hexamer–DDK complex. **g** Resolution of the MCM double hexamer–DDK complex estimated using gold-standard Fourier Shell Correlation. **h** Three rotated views and one cut-through view of the MCM double hexamer–DDK complex 3D structure, color-coded according to the local resolution. Source data

**Extended Data Fig. 3:**
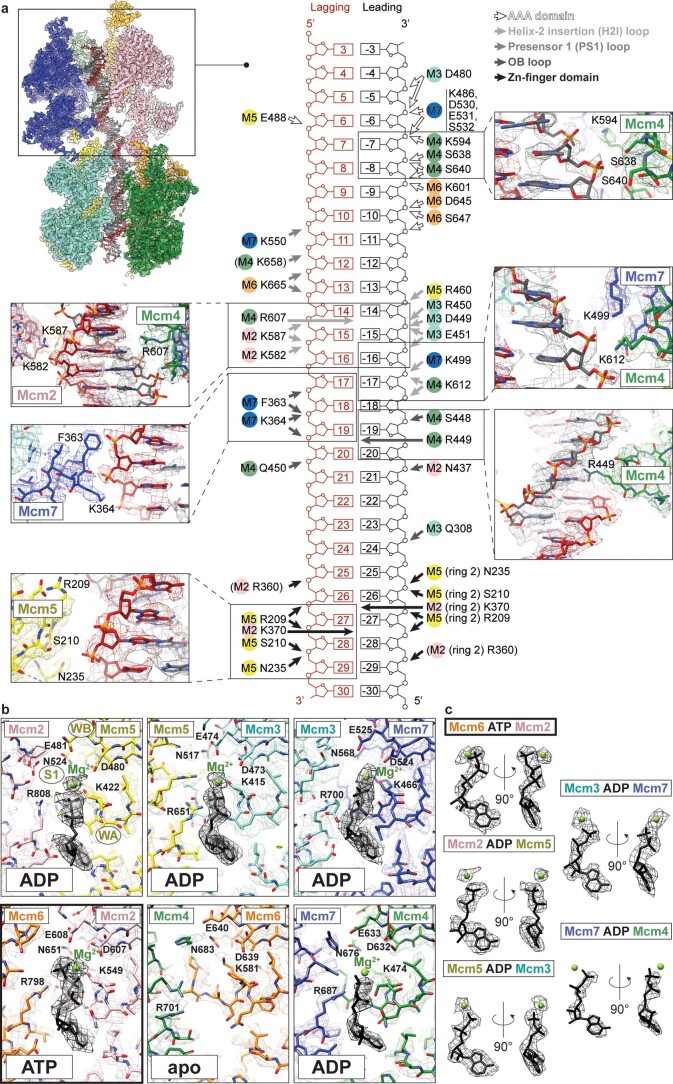
Protein-DNA contact map and illustration of the ATPase sites in the DNA-loaded MCM double hexamer. **a** Model of the residues within the MCM hexamer that contact the DNA. Density and atomic model for selected interactions are shown. **b** Illustration of the nucleotides bound in the different ATPase sites. **c** Segmented density for each of the nucleotides and the coordinated magnesium ion.

To understand DH phosphorylation by DDK, we decided to employ a similar cryo-EM strategy. We imaged ATP-hydrolysis-dependent DH loading on MH-capped ARS1-origin DNA, followed by the addition of the DDK kinase. Two-dimensional (2D) classification revealed previously observed helicase loading intermediates, including ORC-DNA, MCM–Cdt1 and MCM–ORC particles^13,18,19,38^, as well as DHs, alone or in complex with the DDK kinase (Extended Data Fig. 1). Three-dimensional (3D) classification indicated that all DHs were DNA engaged, as expected for MCMs loaded onto a duplex segment capped by covalent roadblocks. After a total of two particle-polishing and eight contrast transfer function (CTF) refinement iterations in RELION^39^, we refined a twofold symmetric, 3.1-Å-resolution structure of DH-DNA, or 3.0-Å resolution after density modification in Phenix^40^ (Fig. 1a,b, Extended Data Fig. 2 and Table 1). At this resolution, individual amino acidic side chains can be visualized contacting specific phosphate groups in the double helix, which allowed us to generate a comprehensive protein-DNA interaction map for the loaded helicase (Fig. 1a and Extended Data Fig. 3). We also obtained an unprecedented view of MCM ATPase centers, allowing us to correct tentative nucleotide occupancy assignments, which were based on lower-resolution data^15,20^. We identified one nucleotide-free site (Mcm4-6) and four adenosine diphosphate (ADP)-bound sites (2-5, 5-3, 3-7 and 7-4), supporting the notion that helicase loading requires ATP hydrolysis by MCM^2,41,42^. We were, however, surprised to observe an ATP molecule harbored within the Mcm6-2 interface (Fig. 1c and Extended Data Fig. 3), given that helicase loading is impaired by an Mcm6 arginine finger mutation that is understood to block ATP hydrolysis^41,42^. Our finding suggests that ADP might be released and a new ATP molecule bound to the Mcm6-2 active site—for example, after loading of a first MCM ring and before DH formation. Another possibility is that ATP binding might be affected by an amino acid change in the Mcm6 arginine finger. Further work will be required to understand the exact role of ATP binding, hydrolysis and ADP release during the sequential loading of a DH.

**Table 1:** Cryo-EM data collection, refinement and validation statistics

	MCM DH	MCM-DDK
	(EMD-13176,	(EMD-13211,
	PDB 7P30)	PDB 7P5Z)
**Data collection and processing**		
Magnification	130,000	
Voltage (kV)	300	
Electron exposure (e^–^/Å^2^)	51.3	
Defocus range (μm)	−2 to −4.1	
Pixel size (Å)	1.08	
Symmetry imposed	C2	C1
Initial particle images (no.)	3,529,085	3,529,085
Final particle images (no.)	238,620	149,876
Map resolution (Å)	2.95	3.3
FSC threshold	0.143	0.143
Map resolution range (Å)	2.7–4.5	3.0–8.5
**Refinement**		
Initial model used (PDB code)	6F0L (MCM-DH-DNA)	6F0L (MCM-DH-DNA)
	5BK4 (MCM-DH-DNA)	5BK4 (MCM-DH-DNA)
	6EYC (MCM-DH-DNA)	6EYC (MCM-DH-DNA)
		6YA7 (Cdc7-Dbf4)
		3QBZ (Dbf4 BRCT)
Model resolution (Å)	3.1	3.6
FSC threshold	0.5	0.5
Map sharpening *B* factor (Å^2^)	ResolveCryo-EM	−10
Model composition		
Nonhydrogen atoms	62,909	67,425
Protein residues	7,766	8,315
Ligands	30 (2 ATP, 8 ADP, 10 Mg^2+^, 10 Zn ^2+^)	31 (2 ATP, 8 ADP, 10 Mg^2+^, 11 Zn ^2+^)
*B* factors (Å^2^)		
Protein	3.37/73.99/27.47	77.38/416.67/133.20
Ligand	8.86/93.94/27.55	102.71/268.16/126.96
R.m.s. deviations		
Bond lengths (Å)	0.003	0.003
Bond angles (°)	0.551	0.568
Validation		
MolProbity score	1.40	1.77
Clashscore	4.34	10.77
Poor rotamers (%)	0.06	0.03
Ramachandran plot		
Favored (%)	96.88	96.62
Allowed (%)	3.08	3.30
Disallowed (%)	0.04	0.09

### DDK engagement explains N-terminal Mcm4 phosphorylation

**Fig. 2:**
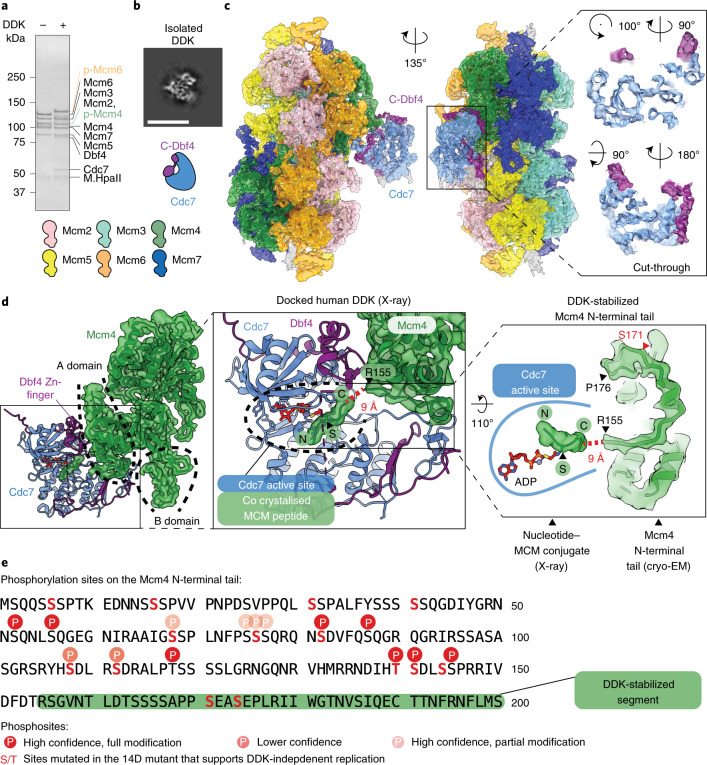
Molecular basis for kinase recognition and phosphorylation of the MCM DH substrate. **a**, SDS–PAGE gel of unmodified and DDK-phosphorylated MCM DHs, tethered to DNA beads. The phosphorylation-dependent shifts of Mcm4 and Mcm6 are highlighted in green and orange, respectively. Notably, after a low-salt-wash step, DDK remains bound to the MCM. Representative of at least *n* = 3 independent experiments. **b**, 2D class average of the isolated DDK shows subnanometer-resolution features, indicating that DDK is a suitable cryo-EM target. Scale bar, 10 nm. **c**, 3.3-Å-resolution structure of the MCM DH–DDK complex, showing the catalytic core of DDK (Cdc7 bound to C-terminal Dbf4), engaged to the Mcm4 subunit of one MCM ring in the DH. Two cut-through views of the kinase core are shown to highlight how the atomic model matches cryo-EM density. **d**, DDK docks onto the Mcm4 A domain via the Dbf4 zinc finger C domain, and onto the Mcm4 B domain via the Dbf4 M domain. Middle: active site of the human DDK crystal structure, which was cocrystallized with an MCM substrate peptide in the active site of Cdc7. The first resolved N-terminal residue (R155) of Mcm4 in the cryo-EM map neatly aligns with the C-terminal end of the MCM peptide. The Mcm4 N-terminal tail in our structure is therefore suitably poised for phosphorylation by the Cdc7 active site. An N-terminal Mcm4 segment (P155 to R176), which is invisible in the absence of DDK, is partially stabilized in the DH–DDK complex. **e**, One known DDK phosphosite in Mcm4 (S171) maps within the DDK-stabilized N-terminal segment visible in our structure. Five additional known phosphosites and others detected by mass spectrometry map upstream of the modeled N-terminal region of Mcm4. This agrees with the notion that active site access requires extended structural flexibility of the phosphorylation substrate. Sites reported to be important for recruitment of the firing factor Sld3 are highlighted. The uncropped gel image for **a** is available as Source data with the paper online. Source data

Double hexamer loading onto origins in vitro, followed by incubation with DDK, results in activation of MCM phosphorylation as established by SDS–polyacrylamide gel electrophoresis (PAGE) analysis of DNA-affinity-purified origin-loaded DHs. Figure 2a shows signature shifts of MCM subunits 6 and 4 following DDK phosphorylation. Given that the isolated DDK component could be imaged at subnanometer resolution, as judged by 2D averaging analysis (Fig. 2b and Extended Data Fig. 1), we gained confidence that 3D analysis of the DH phosphorylation reaction could be achieved. Because neither crosslinking nor nucleotide analogs were employed in the preparation of DNA-loaded, DDK-treated DHs, we assumed that any resulting cryo-EM structures would reflect the loaded helicase captured during or immediately after phosphorylation.

**Extended Data Fig. 4:**
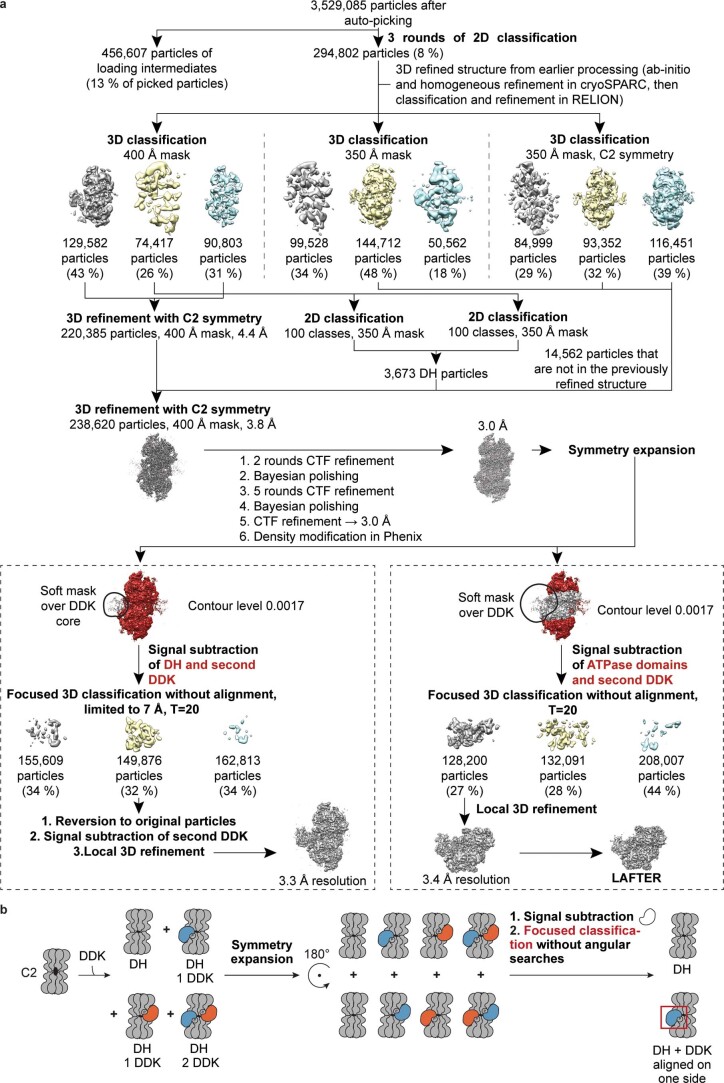
Image processing for the MCM double hexamer–DDK complex. **a** Processing pipeline. **b** Illustration of the symmetry expansion protocol. Partial DDK occupancy on the two-fold symmetric MCM double hexamer hampers structure determination of a DDK–MCM double hexamer complex. To align asymmetrically bound DDK molecules, symmetry expansion was performed. According to this procedure, a copy of the dataset was appended to the MCM–DDK particles, after rotation around the C2 symmetry axis. The signal of one DDK molecule was subtracted from the rotated images to prevent particles from reverting to the original orientation. DDK-bound double hexamer particles were identified by using focused 3D classification skipping angular searches.

One DDK was identified bound to a DH while DH averages bound to two DDK particles were fuzzy, indicating partial occupancy (Extended Data Fig. 1). It was therefore not surprising that C2-symmetrized refinement of the DDK-bound DH revealed poorly defined DDK densities. To improve the DDK features in our reconstruction we performed symmetry expansion^43^. During this process, particles contributing to the twofold symmetrized DH structure were rotated by 180° around the C2 symmetry axis and appended to the original particle stack (Extended Data Fig. 4). To identify DH particles engaged by DDK, we performed 3D classification focused around one DDK feature. We then performed signal subtraction of the symmetry-related DDK subcomplex and refined an asymmetric structure of DNA-DH bound to one DDK complex, to an average resolution of 3.3 Å (Table 1 and Extended Data Fig. 4). A recognizable DDK feature maps peripherally in the structure and is only tenuously tethered to the DH, via three spatially distinct interaction elements (Fig. 2c). Despite limited stability, we were able to reconstruct DDK to a local resolution of ~5 Å (Extended Data Fig. 2), which is sufficient to resolve secondary structure elements within structured domains. Our attempts to perform multibody refinement^44^ or other structural variability analysis did not improve cryo-EM density further, probably due to the inherent flexibility of the kinase particle. This observation reflects the notion that, once DH engaged, DDK phosphorylates several distinct target sites along its target MCM subunit, which presumably results in a continuum of structural conformations.

To build an atomic structure of DNA-DH-DDK, we generated a homology model for yeast DDK based on the crystal structure of the human complex (PDB entry 6YA7)^35^. Following molecular dynamics flexible fitting with Namdinator^45^, manual adjustments in Coot^46^ and further real-space refinement in Phenix^47^, we obtained an atomic model that describes the molecular interaction between the DDK core particle and Mcm4.

**Extended Data Fig. 5:**
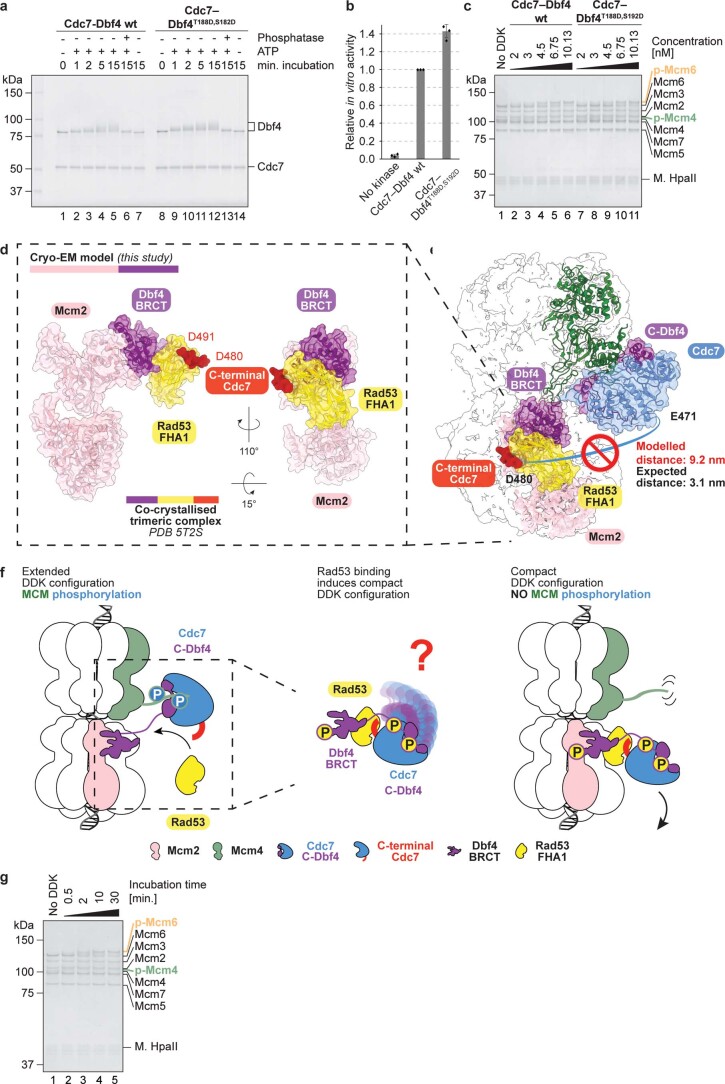
DDK inhibition by Rad53. **a** Phospho-mimicking mutations of T188/S192, which map at the Dbf4–Mcm2 interface, do not interfere with the auto-phosphorylation efficiency of DDK. **b** Quantification of the kinase assay comparing wild type and phospho-mimicking DDK. The phospho-mimicking mutant efficiently phosphorylates the MCM substrate peptide. **c** MCM double hexamer phosphorylation by Cdc7–Dbf4^T188D,S192D^ is comparable to the wild type kinase. **d-f** A possible mechanism of phosphorylation-independent inhibition of DDK by Rad53. **d** The crystal structure of Rad53(FHA1)/Dbf4(BRCT)/Cdc7 (PDB entry 5T2S) superposed to DH–DDK shows no clash between Mcm2 and Rad53. **e** Modelled configuration would not support Mcm4 phosphorylation in *trans*. **f** Model for Rad53 engagement sequestering DDK in a *cis* configuration. Rad53 has been reported to interfere with DH phosphorylation by DDK via two mechanisms, a recently discovered antagonistic binding of Rad53 to DDK that can prevent DH engagement^29^, and the direct phosphorylation of DDK, which blocks late origin firing^11,53^. Our DDK-DH-DNA structure informs both molecular mechanisms (Fig. 5a). To address the phosphorylation-independent, antagonistic binding, we integrated our new cryo-EM data with the published crystallographic information on the Rad53-DDK interaction. The Dbf4 BRCT domain is known to interact with the non-catalytic Forkhead-associated 1 (FHA1) domain of Rad53. When we superposed BRCT-FHA1 co-crystal structure (PDB entry 5T2F) to our DNA-DH-DDK structure we detected no steric clash between FHA1 and the Mcm2 A domain, indicating that FHA1 engagement could still support BRCT docking onto Mcm2. However, when using the structure of a ternary complex, with the FHA1 domain simultaneously bound to Dbf4 and a Cdc7 peptide, we concluded that Rad53 binding would be incompatible with Mcm4 phosphorylation by DDK acting in *trans*. In fact, the Rad53-interacting peptide of Cdc7 (residues 480-491, including phospho-Thr484) maps only 10 residues downstream of the C-terminal end of the Cdc7 atomic model in our DH-DDK structure. A stretch of 10 residues would cover a distance no longer than 3.1 nm, which is shorter than the 9.2 nm distance modelled between the FHA1-engaged Cdc7 peptide and the Cdc7 C-terminus in our DH-DDK atomic model. Therefore, based on our analysis, concomitant binding of Dbf4 and Cdc7 by FHA1 Rad53 could cause the DDK particle to fold back onto itself. When sequestered by Rad53, DDK would be impaired from visiting the extended state that straddles across the two helicase rings. Blocking Mcm4 engagement in *trans* would prevent DH recognition, in turn impairing any activating phosphorylation. However, antagonistic binding of Rad53 to DDK for blocking DH phosphorylation, independent of Rad53 catalytic activity, would require a stoichiometric interaction between the two kinases. This explains why the Rad53-phosphorylation dependent block of origin firing is more readily detected^29,53^. Source data

A conserved zinc finger motif in the Dbf4 C domain engages the Mcm4 A domain, and the intertwined M-KI2 domains from Dbf4-Cdc7 engage the Mcm4 B domain. Thus, the structured Dbf4 elements that wrap around Cdc7 mediate the interaction between the kinase and Mcm4 subunits (Fig. 2d). DDK binding to Mcm4 stabilizes the Mcm4 N-terminal tail that is targeted by Cdc7. Compared to previously published structures^15,20,48^, the Mcm4 subunit could be extended from N-terminal residue 177 to residue 155 in the DDK-engaged model (Fig. 2d). The N-terminal end of the Mcm4 atomic coordinates precedes a stretch of DDK phosphorylation sites that are recognized by Sld3 en route to CMG assembly and support origin activation (Fig. 2e)^26,30,31^. Visible cryo-EM density for the Mcm4 phosphorylation substrate is missing within the active site. This could be due either to the structural flexibility required to facilitate kinase access to multiple N-terminal Mcm4 sites, to some particles being in a post-phosphoryl transfer complex or to a combination of the two. Despite these limitations, assembling a chimeric model that contains our yeast DH and the human DDK structures^35^ can further inform the phosphorylation mechanism. Here, the N-terminal end of the yeast Mcm4 model appears suitably aligned with the C-terminal end of the phosphorylated MCM peptide cocrystallized with human DDK, with the two termini mapping only 7 Å apart in the chimeric structure (Fig. 2d). From our modeling, we conclude that Dbf4-mediated DDK engagement of the A and B domains of Mcm4 positions Cdc7 in a suitable configuration for the phosphorylation of N-terminal Mcm4. Our structure explains the molecular basis of Mcm4 rather than Mcm6 phosphorylation and invites the prediction that Mcm4 phosphorylation of DNA-loaded DHs could be more efficient compared to Mcm6. In agreement with this notion, a gel-based DH phosphorylation titration assay indicated that complete phosphodependent Mcm4 shifting requires threefold lower DDK concentration compared to Mcm6 (Fig. 4c). Likewise a gel-based, time course assay for DHs incubated with 10 nM DDK showed complete phosphodependent Mcm4 shifting after 10 min, but only partial Mcm6 phosphorylation after as long as 30 min (Extended Data Fig. 5). Further supporting the relevance of our Mcm4-engaged DDK structure, we note that Mcm4 phosphorylation alone is sufficient to support origin activation, either in vitro or in cells^24,26,29–32^.

**Fig. 3:**
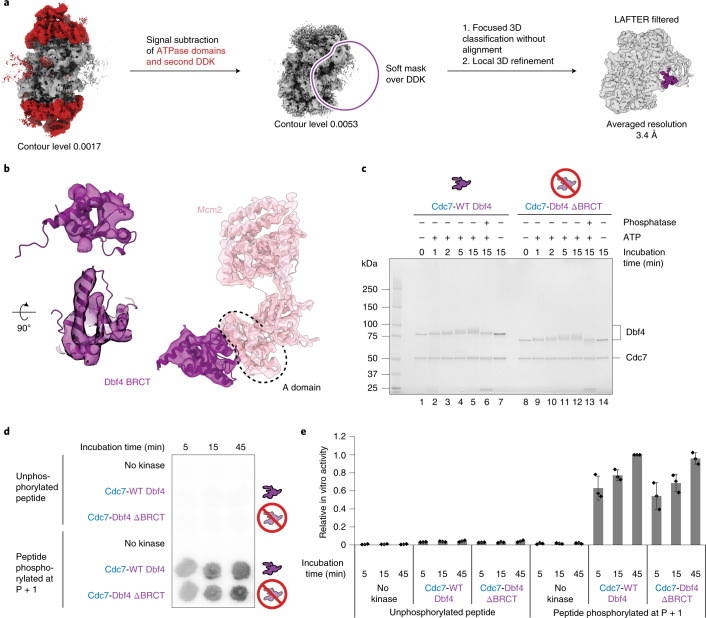
The Dbf4 BRCT domain docks onto the A domain of Mcm2 and its truncation does not affect Cdc7 catalytic activity. **a**, Signal subtraction of the MCM ATPase domains, followed by focused classification, 3D refinement and LAFTER filtering, allowed resolution of the docking site of DDK (shown in purple) onto the MCM DH. **b**, The crystal structure of the Dbf4 BRCT domain (PDB entry 3QBZ) was fitted to the newly resolved docking site, which contacts the A domain of the Mcm2 subunit. **c**, Wild-type (WT) DDK (lanes 1–7) and DDK containing a Dbf4 BRCT truncation (lacking residues 119–219, lanes 8–14) show comparable autophosphorylation efficiency, which can be reverted by lambda phosphatase treatment (lanes 7 and 14). Representative of at least three independent experiments. **d**, Autoradiograph of a kinase assay using a well-characterized substrate of DDK kinases (residues 35–47 of human Mcm2). **e**, Quantification of the kinase assay. The average of three biological replicas is plotted and error bars show s.d. Reads were normalized to the 45-min time point of wild-type DDK. The DDK mutant shows wild-type levels of MCM phosphorylation. As described for the human ortholog, phosphorylation by Cdc7 requires prephosphorylation of Ser41 (P + 1). Uncropped gel images for **c** and **d** are available as Source data with the paper online. Source data

**Fig. 4:**
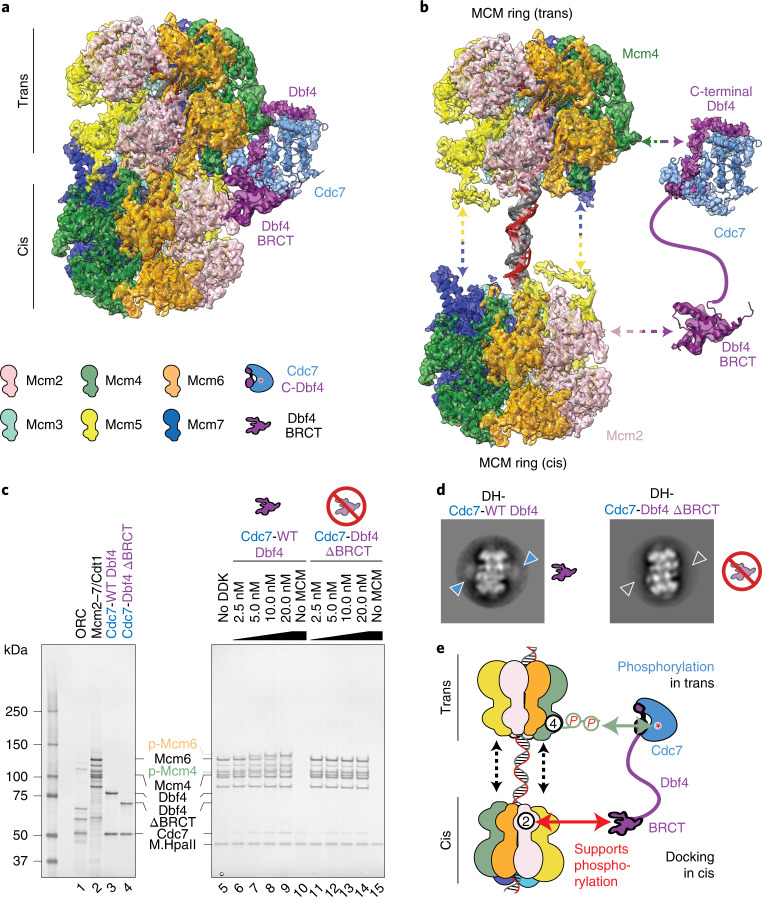
DDK docking onto the MCM DH in cis is required for MCM phosphorylation in trans. **a**,**b**, Composite map of DH structure bound by the DDK core particle and LAFTER-filtered BRCT Dbf4 density shows an extended configuration adopted by DDK, with docking onto Mcm2 in cis and phosphorylation of Mcm4 in trans; composite map and atomic model (**a**). The network of protein contacts that support DH phosphorylation by DDK is illustrated by artificial separation of the different interactors (**b**). **c**, Titration of wild-type DDK shows efficient phosphorylation of DNA-loaded MCM DHs (lanes 6–9). When the ΔBRCT DDK mutant was tested in the same concentration range, no phosphorylation was detected. The effect was observed in three independent experiments. **d**, Negative-stain 2D class averages of MCM DHs incubated with wild-type or ΔBRCT DDK. In the absence of the Dbf4 BRCT docking domain, no DDK density can be visualized bound to the MCM DH. **e**, Model illustrating the importance of DDK docking in cis for MCM phosphorylation in trans. Source data

### Dbf4-mediated docking onto Mcm2

We established that the core DDK particle contacts Mcm4. However, additional, less defined density could be identified clustered around the A domain of Mcm2. This observation is notable given that Mcm2 has previously been identified as a docking site for DDK^29,49^. To improve cryo-EM density adjacent to Mcm2, we performed signal subtraction of the two C-terminal ATPase tiers in the DH, in an attempt to minimize structural flexibility in the aligned core particle. We then performed focused classification on the full DDK density, encompassing both the Mcm2- and Mcm4-interacting elements. After performing LAFTER filtering^50^ to denoise the refined 3.4-Å-resolution structure (Fig. 3a), we recovered Mcm2-interacting density of sufficient quality to unequivocally fit the crystal structure of the yeast Dbf4 BRCT domain (Fig. 3b) (residues 119–219, PDB entry 3QBZ)^34^. Our result reveals that BRCT mediates DDK docking onto the DH by contacting the A domain of Mcm2. This observation also agrees with the notion that a C-terminal deletion mutant containing amino acids 1–320 is sufficient for Dbf4 recruitment to the ARS1 origin in cells^51^.

### DH phosphorylation by DDK occurs in trans

The Dbf4 BRCT domain is connected to the DDK core particle via a 40-amino-acid flexible linker. This structural flexibility allows DDK to visit an extended configuration, where Dbf4 BRCT touches Mcm2 on one ring (which we refer to as in cis) while Cdc7 phosphorylates the juxtaposed Mcm4 in trans, as observed in a composite map that combines LAFTER-filtered BRCT Dbf4 density with the DH structure bound by the DDK core particle (Fig. 4a) or depicted in the interactome map (Fig. 4b). Such architecture immediately suggests a mechanism for how DDK derives specificity for the DNA-loaded DH, by recognizing its 3D shape. To test our model, we generated a DDK variant containing an internal truncation of the Dbf4 BRCT motif (amino acids 119–219, hereafter ΔBRCT). We first asked whether the BRCT truncation affects kinase activity, by taking two distinct approaches. (1) We performed a DDK autophosphorylation time course experiment. Both wild-type and ΔBRCT displayed similar phosphorylation profiles, as established by the appearance of a phosphorylation-dependent shift in the Dbf4 and Cdc7 bands in a silver-stained protein gel, which can be reverted by lambda phosphatase treatment (Fig. 3c). (2) We tested our proteins in a time course kinase assay using a well-characterized DDK substrate (residues 35–47 of human Mcm2), which contains target residue Ser40 and is primed by phosphorylation on Ser41 (ref. ^52^). Both wild-type DDK and ΔBRCT displayed negligible phosphorylation of the unmodified peptide and showed equally robust phosphorylation following phospho-Ser41 priming, as previously observed for human DDK^33,35,52^ (Fig. 3d,e). We conclude that the BRCT truncation of Dbf4 has no effect on DDK enzymatic activity by itself. We next asked whether the BRCT truncation had any effect on DH phosphorylation by DDK. To address this question, we phosphorylated DHs loaded onto MH-roadblocked origin DNA and tethered to streptavidin beads. Phosphorylation-dependent shifts of Mcm4 and Mcm6 were detected when DDK was titrated between 2.5- and 20-nM concentration. When assayed within the same concentration range, ΔBRCT caused no detectable phosphorylation of either Mcm4 or Mcm6 (Fig. 4c), nor was any ΔBRCT binding to DH observed when the same reaction was visualized by negative-stain EM (Fig. 4d). Our results suggest a mechanism whereby DDK recognizes loaded DHs by docking onto the A domain of Mcm2 via the BRCT domain of Dbf4 and phosphorylates the Mcm4 in trans by recognizing the A and B domains via the Dbf4 M and C domains (Fig. 4e).

### Selective phosphorylation of DNA-loaded MCM DHs

According to our model, the geometry of MCM engagement by DDK facilitates the recognition of critical Mcm4 target residues in the loaded DH but would not do so in the loading-competent, monomeric MCM–Cdt1 complex. To test our hypothesis, we identified biochemical conditions to achieve MCM–Cdt1 phosphorylation (robust, yet less efficient than DH phosphorylation), which involved increasing the kinase/target molar ratio by ~sevenfold. We used mass spectrometry analysis to compare the MCM–Cdt1 post-translational modification profile with that of the DNA-loaded DH (Supplementary Table 1). While the Mcm4 subunit was phosphorylated on N-terminal serine residues S52 and S56 in both complexes, only DHs were phosphorylated on previously reported DDK sites^24^, including S76, S77 and S87, which are known to be read by the firing factor Sld3 and support origin activation both in vitro^30^ and in vivo^31^. Mcm6 followed a similar pattern, with Thr75 and Ser78 being selectively phosphorylated in the DH (Supplementary Table 2). From our mass spectrometry analysis, we conclude that DH recognition by DDK drives both the efficiency and specificity of the phosphorylation reaction.

**Fig. 5:**
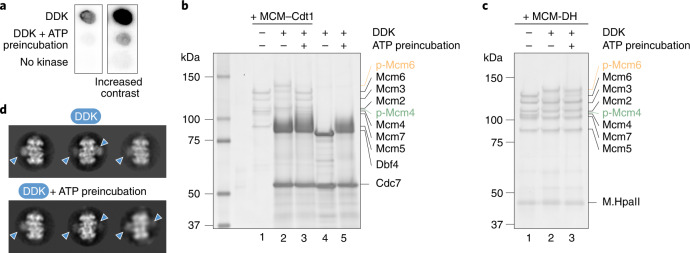
DDK preautophosphorylation prevents peptide and MCM–Cdt1 phosphorylation, but not DH phosphorylation and engagement. **a**, MCM peptide phosphorylation drops by 92% following DDK preautophosphorylation. **b**, MCM–Cdt1 phosphorylation is abrogated following DDK preautophosphorylation. **c**, DH phosphorylation is virtually unperturbed following DDK preautophosphorylation. **a**–**c**, Representative images of three independent experiments. **d**, 2D averages derived from negatively stained particles indicating that DDK binding to DHs is unperturbed following DDK preautophosphorylation. Uncropped images for **a** and **c** are available as Source data with the paper online. Source data

We then wondered whether DDK autophosphorylation, which is known to modulate its kinase activity, could specifically affect substrate selectivity. The results we obtained were surprising. We preincubated DDK with ATP to achieve robust autophosphorylation and used this reagent in kinase experiments with a range of MCM substrates. We found that DDK autophosphorylation induces a 92% decrease in the efficiency of MCM peptide phosphorylation in vitro (Fig. 5a) and completely abolishes MCM–Cdt1 phosphorylation (Fig. 5b). However, phosphorylation efficiency of the DNA-loaded DH is virtually unperturbed when using unmodified or preautophosphorylated DDK (Fig. 5c). Likewise, DH engagement by DDK was unaffected by preautophosphorylation, as detected by 2D averaging of negatively stained particles (Fig. 5d). Our findings agree with the observation that preautophosphorylation of DDK has no effect on replication origin firing in vitro^53^. Collectively, these data support the notion that optimal substrate engagement achieved via DDK docking onto DHs overrides the inhibitory effect derived from autophosphorylation. Thus, autophosphorylation increases DDK kinase fidelity towards DNA-loaded helicases poised to support bidirectional replication.

### Mechanism of phosphodependent inhibition of DDK by Rad53

**Fig. 6:**
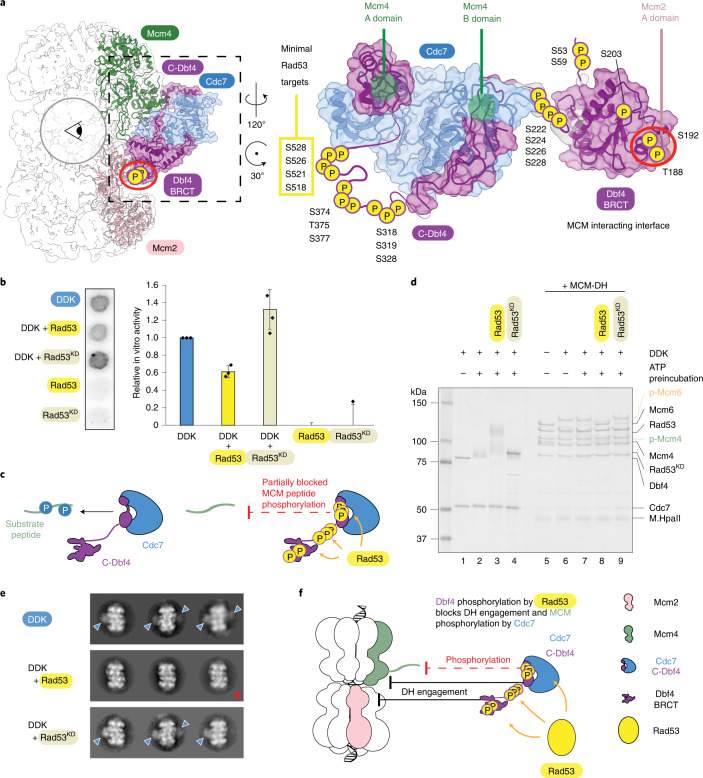
DDK inhibition by Rad53. **a**, Rad53 phosphotargets on Dbf4. S518–S528 phosphorylation blocks origin firing in cells. **b**, Autoradiograph and quantification of a kinase assay comparing peptide phosphorylation after treatment of DDK with Rad53 or an enzymatically inactive version (Rad53^KD^). The average of three biological replicas is plotted and error bars shows s.d. **c**, Model showing how phosphorylation by Rad53 interferes with the Cdc7 active site, independent of DH engagement. **d**, Phosphorylation of DNA-loaded MCM DHs is inhibited after Rad53-dependent phosphorylation of DDK, but not by the presence of Rad53^KD^. The effect was observed in three independent experiments. **e**, Table summarizing engagement of DDK with MCM DHs in the presence of Rad53 and Rad53^KD^ when imaged by negative-stain EM. Representative 2D class averages of DHs and DDK-engaged DHs are shown from 6,033 (no Rad53), 7,018 (WT Rad53) and 5,519 (kinase-dead Rad53) picked DHs. Blue arrowheads indicate DDK bound to the DH and the red cross indicates DH without DDK. **f**, Model showing how Rad53-dependent phosphorylation of Dbf4 prevents DDK from docking onto the MCM and inhibits MCM phosphorylation. Uncropped images for **b** and **d** are available with the paper online. Source data

It is established that Dbf4 phosphorylation by Rad53 blocks late origin firing via impairment of DH phosphorylation by DDK; however, the molecular mechanism is not understood^53^. To gain structural insights into this regulatory process, we mapped the 19 known Rad53 phosphorylation targets^11^ on our DDK-DH structure and identified two classes of residue. In one class, phosphosites reside on the structured BRCT domain while in the other they map in flexible loops (not modeled in our structure) that connect the BRCT, M and C domains of Dbf4 (Fig. 6a). Several phosphosites cluster on the MCM-interacting side of Dbf4, raising the question of whether phosphorylation by Rad53 affects the Cdc7 active site or, rather, globally interferes with DH recognition by DDK.

To test these hypotheses, we began by recapitulating the published observation that preincubation of DDK with Rad53 and ATP abrogates DH phosphorylation in vitro^29^. We found that this effect is dependent on phosphorylation by Rad53, given that the catalytically dead Rad53 showed no inhibition of DH phosphorylation by DDK^53^. We asked whether impairment of DH phosphorylation occurs because Rad53 interferes with the Cdc7 active site, independent of DH recognition. To address this issue we employed the peptide phosphorylation assay introduced above. Compared to kinase activity levels detected for DDK preincubated with ATP, preincubation of DDK with Rad53 and ATP resulted in a 40% reduction in MCM peptide phosphorylation (Fig. 6b,c and Extended Data Fig. 5), which is substantial although distinct from the complete abrogation of MCM modification observed in the DH phosphorylation assay (Fig. 6d). This inhibitory effect appears to be due to DDK phosphorylation by Rad53, given that preincubation with a catalytically dead variant showed no evident reduction in DH phosphorylation by DDK under the experimental conditions tested. Neither wild-type nor mutant Rad53 phosphorylated the MCM substrate in our peptide-based assay, confirming the notion that the target of Rad53 phosphorylation is DDK. We note that, amongst the 19 known Rad53 phosphosites on Dbf4, the minimal targets critical for checkpoint inhibition (S518, S521, S526 and S528) reside on a loop (not resolved in our structure) that would map most closely to the Cdc7 catalytic center and could either gate MCM-tail access or directly affect active site structure and functionality. In summary, our findings indicate that Dbf4 phosphorylation by Rad53 has a substantial inhibitory effect on the Cdc7 active site, athough it does not completely impair MCM phosphorylation.

To test the hypothesis that Rad53 phosphorylation blocks DH recognition by DDK, we took two distinct approaches. First, we identified two Rad53 target residues that might play a critical role in DDK docking onto the DH. One is Thr188, which is buried within the Dbf4 BRCT and Mcm2 interaction interface, and the other is Ser192, mapping in close proximity. To test whether modification of these two sites is sufficient to block DH docking and phosphorylation efficiency by DDK, we generated a DDK variant containing two phosphomimicking amino acid changes, T188D/S192D. The double mutant presented wild-type levels of kinase activity, as established using autophosphorylation and peptide phosphorylation assays. When probed in a DH phosphorylation experiment, the same variant also displayed wild-type behavior across a range of DDK concentrations (Extended Data Fig. 5). Likewise, DH decoration by the T188D/S192D DDK variant was similar to that of the wild-type protein as established by negative-stain 2D averaging (not shown). Our observations suggest that phosphorylation of the DDK docking site is insufficient to block the interaction with Mcm2 and MCM phosphorylation in trans. This notion agrees with the observation that T188 and S192 fall outside of the shortlist of four Rad53 target sites (minimal Rad53 targets, Fig. 6a), whose change to alanine bypasses Rad53-dependent checkpoint regulation in cells^11^. The second approach to test the effect of Rad53 phosphorylation on DH engagement by DDK involved negative-stain EM imaging of DH phosphorylation reactions with or without preincubation of DDK with Rad53 and ATP. We found that DDK preincubation with wild-type, but not with catalytically dead Rad53, completely abrogates DH engagement by DDK (Fig. 6d).

Collectively, our data indicate that Rad53 blocks DDK phosphorylation of DHs by multiple mechanisms. It affects the Cdc7 active site, as established by the 40% reduction in phosphorylation efficiency in our peptide-based assay. It also directly affects DH engagement by DDK. In this process it is unlikely that individual, Rad53-mediated phosphorylation would play a major role in blocking this interaction. Rather, inhibition is probably due to the cumulative effect of the 19 Rad53 phosphosites spanning the length of the Dbf4 molecule.

## Discussion

Bidirectional replication in eukaryotes starts when the paths of two helicase rings cross as they translocate along single-stranded DNA, with their N-terminal domain forming the leading edge of the advancing replication machinery. Through this mechanism, the DNA strand excluded from the central pore of one helicase must necessarily become the translocation strand of the second helicase and vice versa. This ensures that two diverging replication forks are always established upon firing of one origin of replication, exposing single-stranded DNA for replicative polymerases. The symmetry of bidirectional replication is defined upon loading a pair of six-membered MCM rings onto duplex DNA that form an N-to-N DH^3,5,15,20^. The first step towards switching on the DNA unwinding function in MCM is phosphorylation of the DNA-loaded form of the helicase by DDK^7^. It is assumed that only activation of DHs will establish bidirectional replication. In fact, DDK adds activating phosphorylation marks to DHs but not to other MCM loading intermediates^24^. How DDK can selectively phosphorylate this fully loaded MCM species remains an outstanding question in this field. Our discovery that DDK docks onto Mcm2 in one ring and phosphorylates Mcm4 on the opposed ring resolves this mystery. In fact, the observation that docking in cis is essential for phosphorylation in trans reveals that selectivity of the kinase target derives from recognition of the 3D shape of the loaded DH. Through this mechanism DDK can count the number of rings loaded onto origin DNA and ensure that their relative orientation is suitable to support bidirectional replication, as the paths of the two N-to-N interacting rings are poised to cross.

Our cryo-EM structure also describes additional DH interaction elements in the Dbf4 subunit of DDK, explaining why their mutation affects cell viability. One example is the Zn-Finger C domain, which engages the A domain of Mcm4 (ref. ^33^). A second feature is formed by the Cdc7-KI2 activation loop element intertwined with the M domain of Dbf4, which modulates kinase function^35^. This feature projects from the Cdc7 core module to contact the B domain of Mcm4, contributing to stabilization of the kinase to its target subunit. In fact, these interactions orient the active site of Cdc7, enabling the phosphorylation of N-terminal Mcm4 which, in turn, initiates a cascade of events leading to origin activation^7^.

Our structure provides insights into the notable observation that a point mutation in the Mcm5 subunit (a L83P substitution, known as Bob1 mutation) bypasses the requirement for Cdc7 phosphorylation in yeast cells^54^. In fact, we discovered that when Mcm4-engaged the core DDK particle becomes nestled in a cavity between the Mcm4 and Mcm5 A domains, which are juxtaposed at the dimerization interface in the DH. It can be envisaged that the Bob1 change in Mcm5 might cause a structural alteration that prevents DDK binding to the Mcm4/5 cavity while promoting Sld3/7 recruitment to the same Mcm4/5 site, independent of Mcm4 phosphorylation. This would in turn drive the recruitment of Cdc45 en route to CMG formation^30,55^. Structural work on the Bob1 change introduced in an archaeal MCM ortholog supports this notion^56^.

Although Mcm4 phosphorylation is sufficient to drive origin activation, concomitant mutation of Mcm4 and Mcm6 sites is required for observable growth phenotypes^24,26,29–32^. How Mcm6 becomes phosphorylated following DDK docking onto Mcm2 is not addressed by our data, but two possible scenarios can be envisaged. In one, the DDK kinase core maintains an interaction with Mcm4 while the N-terminal tail from the neighboring Mcm6 subunit snakes into the Cdc7 active site. In the second scenario, DDK maintains the Mcm2 docking interaction in cis but swaps from engaging Mcm4 to binding Mcm6 in trans.

Not only does our work inform the structural mechanism for DH phosphorylation by DDK, it also explains how the checkpoint kinase Rad53 regulates origin activation by impairing late origin firing when DNA damage is detected. Although it is known that Rad53 blocks DH phosphorylation by DDK via Dbf4 modification, the mechanism is unclear^8,11^. Led by our structural findings, we performed experiments that uncovered a redundant mechanism, which combines interference with Cdc7 activity, as well as impairment of DDK engagement onto DNA-loaded DHs. We found that Rad53 phosphorylation reduces DDK kinase activity by 40% when tested outside the context of DH engagement in an MCM peptide phosphorylation assay. Under the same conditions, however, DH phosphorylation is completely abrogated, suggesting that Rad53 phosphorylation additionally affects DH binding by DDK. Negative-stain EM analysis confirms this hypothesis, by showing that Rad53 phosphorylation blocks DH decoration by DDK. We find that the inhibitory effect is dependent on the kinase activity of Rad53, because a catalytically defunct variant does not affect DDK function. This is in line with the observation that the same Rad53 variant fails to impair origin activation in vitro^53^. Blocking DH phosphorylation probably requires the combined effect of several Rad53 phosphorylations on Dbf4. In fact, the introduction of two phosphomimetic mutations on the Mcm2-interacting surface of the Dbf4 BRCT domain (acting in cis, ring 1) fails to abrogate DH engagement and phosphorylation by DDK. This suggests that targeting both cis- (Mcm2 interactors, ring 1) as well as trans-acting elements (Mcm4 interactors, ring 2) is required to block DH recognition. A third mechanism for Rad53 inhibition of DDK has been proposed, which does not rely on Rad53 kinase activity but rather on the antagonistic binding of Rad53 to DDK that prevents DH engagement^29^. While we were unable to detect any inhibitory effect of kinase-dead Rad53 in our peptide, DH phosphorylation or DH engagement EM assays, our cryo-EM structure allows us to rationalize how DDK sequestration by Rad53 might work (Extended Data Fig. 5).

In summary, we describe the mechanism whereby DDK selectively targets fully loaded MCM DHs on the path to helicase activation, and uncovered the mechanism whereby the checkpoint kinase Rad53 blocks this function. DDK phosphorylation is only the first step towards assembly of the active CMG replicative holohelicase that promotes origin firing^7^. Future work will establish whether other firing factors form contacts across the DH like DDK, to selectively deposit helicase activators onto fully loaded MCM particles that support bidirectional replication. The existence of single-loaded MCMs has recently been detected in single-molecule fluorescent studies on wild-type proteins^57^, while a further report describes an MCM mutant that can be loaded onto origins but fails to form DHs or promote replication fork establishment^58^. An interesting question for the field will be describing the role of the symmetric DH complex in recruitment of the multiple firing factors needed to activate DNA unwinding in the MCM motor, downstream of DDK phosphorylation.

## Methods

### Construction of yeast expression strains

#### Cdc7-CBP-Dbf4Δ119-219

To generate a yeast strain overexpressing CBP-tagged (ΔBRCT)Dbf4 and wild-type Cdc7, plasmid pRS304 (pRS304/Cdc7-Gal-CBP-Dbf4)^59^ was modified to delete the sequence encoding amino acids 119–219 of Dbf4. The resulting plasmid, pJG1, was integrated into the genome of yeast strain yJF1a to generate strain yJG3.

#### Phosphomimetic mutant of Cdc7-CBP-Dbf4^T188D,S192D^

Codons for T188 and S192 of Dbf4 were simultaneously replaced by codons for aspartate, resulting in plasmid pJG9. The plasmid was integrated into yJF1a, generating strain yJG13.

### Protein expression and purification

#### Expression of ORC, Mcm2-7-Cdt1 and DDK

ORC, Mcm2-7-Cdt1 and DDK were expressed in *Saccharomyces cerevisiae* cells and purified as previously described^17,41,59^. Briefly, all yeast strains were grown at 30 °C in YP medium supplemented with 2% raffinose. When cultures reached 2–3 × 10^7^ cells ml^–1^, expression was induced by the addition of 2% galactose for 3 h. Cells were harvested and washed in the respective lysis buffer, then resuspended in lysis buffer at half pellet volume, flash-frozen in liquid nitrogen and crushed using a 6875D Freezer/Mill Dual Chamber Cryogenic Grinderfreezer mill (SPEX SamplePrep) at intensity 15 (six cycles of 2 min of milling with 1 min of rest).

#### Purification of CBP-tagged ORC

Cell powder from 20 l of yeast culture was resuspended in buffer 1 (25 mM HEPES-KOH pH 7.6, 0.05% NP-40, 10% glycerol, 2 mM beta-mercaptoethanol) supplemented with 0.1 M potassium chloride and protease inhibitors. Potassium chloride concentration was increased to 0.5 M before subjecting the lysate to ultracentrifugation at 235,418*g* (45,000 r.p.m.) for 60 min at 4 °C. Cleared lysates were supplemented with 2 mM calcium chloride and incubated for 2 h at 4 °C with 5 ml of Calmodulin Affinity Resin (Agilent) pre-equilibrated in buffer 1 plus 0.4 M potassium chloride and 2 mM calcium chloride. Beads were subsequently washed with 80 ml of buffer 1 supplemented with 0.4 M potassium chloride and 2 mM calcium chloride, and bound proteins were eluted using buffer 1 supplemented with 0.4 M potassium chloride, 2 mM EDTA and 2 mM EGTA. Pooled fractions were concentrated using a 100,000-molecular weight cutoff (MWCO) spin column and subsequently separated on a Superdex 200 16/600 gel filtration column (GE Healthcare) in buffer 1 supplemented with 0.15 M potassium chloride. Peak fractions were pooled and concentrated.

#### Purification of CBP-tagged Mcm2-7-Cdt1

Cell powder from 12 l of yeast culture was resuspended in buffer 2 (45 mM HEPES-KOH pH 7.6, 100 mM potassium acetate, 5 mM magnesium acetate, 0.02% NP-40, 10% glycerol, 2 mM beta-mercaptoethanol) supplemented with protease inhibitors. Lysate was cleared by ultracentrifugation as described above. The supernatant was subsequently incubated for 2 h with 1 ml of Calmodulin Affinity Resin (Agilent) pre-equilibrated in buffer 2 supplemented with 2 mM calcium chloride. Afterwards, beads were washed with 80 ml of buffer 2 supplemented with 2 mM calcium chloride, and bound proteins were eluted using buffer 2 supplemented with 2 mM EDTA and 2 mM EGTA. Pooled fractions were concentrated using a 100,000-MWCO spin column and subsequently separated on a Superdex 200 16/600 gel filtration column (GE Healthcare) in buffer 2. Peak fractions were pooled and concentrated.

#### Purification of CBP-tagged DDK

Cell powder from 6 l of yeast culture was resuspended in buffer 3 (25 mM HEPES-KOH pH 7.6, 400 mM sodium chloride, 0.02% NP-40, 10% glycerol, 2 mM DTT) supplemented with protease inhibitors. Lysate was cleared by ultracentrifugation as described above, supplemented with 2 mM calcium chloride and incubated for 3 h with 2.5 ml of Calmodulin Affinity Resin (Agilent). Beads were washed extensively with buffer 3 supplemented with 2 mM calcium chloride, incubated twice for 15 min at 4 °C with 20 ml of buffer 3 supplemented with 2 mM calcium chloride, 1 mM ATP, 10 mM magnesium acetate, and washed again with buffer 3 supplemented with 2 mM calcium chloride. Bead-bound proteins were treated for 1 h at 4 °C with 2,800 units of lambda phosphatase (NEB, no. P0753L). Subsequently beads were washed with 100 ml of buffer 3 plus 2 mM calcium chloride, and DDK was eluted using buffer 3 supplemented with 2 mM EDTA. Pooled fractions were diluted to reduce sodium chloride concentration to 0.1 M, then subjected to anion exchange chromatography on a 5-ml heparin column (GE Healthcare). Proteins were separated in a 10-CV gradient from buffer 4 (25 mM HEPES-KOH pH 7.6, 0.02% NP-40, 1 mM DTT) plus 0.1 M sodium chloride to buffer 4 plus 1 M sodium chloride. Pooled fractions were concentrated using a 30,000-MWCO spin column and subsequently separated on a Superdex 200 16/600 gel filtration column (GE Healthcare) in buffer 5 (25 mM HEPES-KOH pH 7.6, 0.2 M potassium glutamate, 0.02% NP-40, 1 mM DTT). Peak fractions were pooled and concentrated.

#### Expression and purification of GST-tagged Cdc6

The expression plasmid for GST-tagged *S. cerevisae* Cdc6 (pAM3) was transformed into BL21 (DE3) RIL *Escherichia coli* cells, and expression and purification were carried out as previously described^17^. Briefly, cells were grown at 37 °C in lysogeny broth (LB) medium to an optical density of 0.6 before induction with 0.5 mM IPTG for 5 h at 18 °C. Cells were harvested by centrifugation at 4,552*g* (4,000 r.p.m.) and room temperature for 10 min.

Cell pellets were resuspended in buffer 6 (50 mM potassium phosphate pH 7.6, 150 mM potassium acetate, 5 mM magnesium chloride, 2 mM ATP, 1% triton, 1 mM DTT) supplemented with protease inhibitor tablets and lysed by sonication. Lysate was cleared by centrifugation at 58,545*g* (22,000 r.p.m.) and 4 °C for 20 min. The soluble phase was applied twice to 2 ml of pre-equilibrated Glutathione Sepharose 4B (GE). Beads were washed with 40 ml of buffer 6 plus protease inhibitors and 40 ml of buffer 6. Beads were resuspended in buffer 6 to generate a 50% slurry and incubated at 4 °C overnight with 50 μl of PreScission protease (GE Healthcare). Eluate was mixed with buffer 7 (50 mM potassium phosphate pH 7.6, 5 mM magnesium chloride, 2 mM ATP, 0.1% triton, 1 mM DTT) to bring potassium acetate concentration to 75 mM and incubated for 15 min with 2 g of Bio-Gel HTP Hydroxyapatite (Bio-Rad) powder, which had been hydrated with buffer 7 supplemented with 75 mM potassium acetate. Beads were washed with 10 ml of buffer 7 supplemented with 75 mM potassium acetate and 15% glycerol, then 10 ml of buffer 7 supplemented with 150 mM potassium acetate and 15% glycerol. Protein was eluted using buffer 7 supplemented with 400 mM potassium acetate and 15% glycerol. Eluted protein was dialyzed against buffer 8 (25 mM HEPES-KOH pH 7.6, 10 mM magnesium acetate, 0.02% NP-40, 100 mM potassium acetate, 10% glycerol, 5 mM beta-mercaptoethanol) for around 20 h and finally concentrated using a 30,000-MWCO spin column.

#### Expression and purification of His-tagged Rad53

Rad53 was expressed and purified as described previously^30,53,60^. Briefly, BL21 (DE3) RIL *E. coli* cells were transformed with plasmid pET21b-RAD53 (ref. ^60^) for expression of wild-type Rad53 or pET21b-RAD53^K227A,D339A^-6xHis^53^ for expression of kinase-dead Rad53. Cells were grown at 37 °C in LB medium to an optical density of 0.4 before induction with 1 mM IPTG for 2 h at 37 °C. Cells were harvested by centrifugation at 4,552*g* (4,000 r.p.m.) and room temperature for 10 min. Cell pellets were resuspended in buffer 9 (25 mM HEPES-KOH pH 7.6, 300 mM sodium chloride, 0.02% NP-40, 10% glycerol) supplemented with protease inhibitor tablets, before lysis by sonication. The soluble phase was separated by centrifugation at 58,545*g* (22,000 r.p.m.) and 4 °C for 20 min. The clear phase was supplemented with 10 mM imidazole and incubated for 1 h at 4 °C with Ni-NTA Agarose (Qiagen). Beads were washed with buffer 9 plus 10 mM imidazole. His-tagged Rad53 was eluted with buffer 9 plus 200 mM imidazole. Pooled peak fractions were concentrated using a 30,000-MWCO spin column and separated on a Superdex 200 increase 10/300 gel filtration column (GE Healthcare) equilibrated in buffer 9. Peak fractions were pooled and concentrated.

### DNA template preparation

A 168-base pair linear DNA construct containing the *S. cerevisiae ARS1* origin sequence was flanked by two covalently linked HpaII methyltransferases (M.HpaII, NEB). The 5' ARS1 end was engineered with a desthiobiotin TEG to allow affinity purification. The DNA construct was generated and purified as previously described^13^. Briefly, the origin template was amplified by PCR where each primer contained the modified recognition sequence for M.HpaII (CC*GG) with the suicide substrate 5-fluoro-2'-deoxycytosine (C*). Free oligonucleotides were removed by anion exchange chromatography using a 1-ml Resource Q column (GE Healthcare) with a 24-CV gradient from buffer 10 (50 mM Tris pH 8.0, 5 mM beta-mercaptoethanol) to buffer 7 plus 2 M sodium chloride. Peak fractions were pooled and subjected to ethanol precipitation. Washed pellets were resuspended in TE buffer. To conjugate DNA templates, M.HpaII and DNA were mixed in a 3:1 molar ratio. The reaction was carried out overnight at 30 °C in buffer 11 (50 mM potassium acetate, 25 mM Tris pH 7.5, 10 mM magnesium acetate, 1 mg ml^–1^ bovine serum albumin (BSA), 150 μM S-adenosyl-methionine (NEB)). Products were then separated on a 1-ml Resource Q column and a 40-CV gradient from buffer 10 to buffer 10 plus 2 M sodium chloride. The final product was concentrated using a 30,000-MWCO spin column.

### MCM loading and phosphorylation

For DNA-pulldown assays, 2.25 nM DNA was coincubated with 5.5 nM ORC, 5.5 nM Cdc6 and 5 mM ATP for 10 min at 30 °C in buffer A supplemented with 5 mM ATP with mixing at 1,250 r.p.m. Mcm2-7-Cdt1 was added to a final concentration of 7 nM and incubation was continued for a further 30 min. Per 40-μl reaction, 2 μl of M280 streptavidin paramagnetic beads (Invitrogen) was added and allowed to interact for 30 min with DNA-bound protein complexes. Unbound complexes were removed by washing three times with high-salt buffer (25 mM HEPES-KOH pH 7.6, 500 mM NaCl, 5 mM magnesium acetate, 0.02% NP-40), twice with buffer A and then resuspended in 40 μl of buffer A supplemented 5 mM ATP. DDK was incubated for 15 min at 30 °C in titration experiments. Reactions were washed with low-salt buffer (25 mM HEPES-KOH pH 7.6, 300 mM sodium acetate, 5 mM magnesium acetate, 0.02% NP-40) unless indicated differently. DNA-bound complexes were eluted by the addition of micrococcal nuclease (MNase, NEB, 1,000 units) and incubation for 10 min at 37 °C. Eluted complexes were analyzed by SDS–PAGE and silver staining (SilverQuest Silver Stain, Bio-Rad).

### Autophosphorylation of DDK

DDK (70 nM) was incubated at 30 °C with mixing at 1,250 r.p.m. in 80 μl of buffer A. During the time course, 10 μl of the reaction was removed, mixed with Laemmli sample buffer and heated to 97 °C for 10 min. For treatment with lambda phosphatase, lambda phosphatase (NEB, 10 units) and manganese chloride (1 mM final concentration) were added to 10 μl of the DDK autophosphorylation reaction. Dephosphorylation was carried out for 30 min at 30 °C with mixing at 1,250 r.p.m., before termination of the reaction with Laemmli sample buffer. All reactions were analyzed by SDS–PAGE and silver staining.

### Peptide phosphorylation assay

DDK kinase assays were carried out as previously described^33,35,61^ with several modifications. Briefly, N-terminally biotinylated peptides containing residues 35–47 of human MCM2 (^35^TDALTSSPGRDLP^47^), containing either phosphorylated or unphosphorylated Ser41, were used as substrates for DDK phosphorylation. Next, 27.5 μg of biotinylated peptide was incubated with 2.8 nM DDK and 16.5 μCi of [γ-^32^P]ATP (5,000 Ci mmol^–1^) in 137.5 μl of kinase assay reaction buffer (40 mM HEPES-KOH pH 7.6, 10 mM magnesium acetate, 2 mM DTT, 0.1% NP-40, 80 μg ml^–1^ BSA, 1 mM β-glycerophosphate, 1 mM sodium fluoride, 0.1 mM ATP). The time course experiment was performed at 30 °C. At the indicated time points, 25-μl volumes were removed from each reaction and terminated by the addition of guanidine hydrochloride to a final concentration of 2.5 M, followed by spotting of 12.5 μl onto SAM2 biotin-capture membranes (Promega). The membranes were washed three times with 2 M NaCl, four times with 2 M NaCl in PBS, twice in distilled water and finally in 95% ethanol. Membranes were air-dried and analyzed by phosphorescence imaging with Typhoon FLA 9500 (GE Healthcare).

In the experiment shown in Fig. 4f, 50 nM DDK was autophosphorylated by incubation with 5 mM ATP in 20 μl of buffer A for 15 min at 30 °C, and peptide phosphorylation was performed for 45 min as described above. Phosphorylation of DDK by Rad53 before peptide phosphorylation (Fig. 5b) was performed by mixing DDK and Rad53 at an equimolar ratio (50 nM) and incubation with 5 mM ATP in 20 μl of buffer A for 15 min at 30 °C. MCM peptide phosphorylation was then conducted as described above.

### Negative-stain EM sample preparation

#### DH phosphorylation by wild-type or ΔBRCT DDK

DH phosphorylation by wild-type or ΔBRCT DDK was analyzed using negative-stain EM. To assemble the reaction, 100 nM each of ORC, Cdc6 and Mcm2-7-Cdt1 was coincubated with 50 nM DNA in 10 μl of buffer B for 30 min at 30 °C, and 1,250 r.p.m. DDK was added to a final concentration of 150 nM. After 15 min, samples were diluted to a final protein concentration of 20 ng μl^–1^, 4 μl of phosphorylation reaction was applied to freshly glow-discharged 400-mesh copper carbon grids (Agar Scientific, no. AGS160-4) followed by incubation for 1 min. Grids were stained using four 30-μl drops of 2% uranyl acetate solution with gentle stirring for 10 s each. Excess stain was removed by blotting with filter paper.

#### Rad53 inhibition of DH phosphorylation

MCM DHs were assembled on roadblocked DNA by coincubation of 100 nM DNA with 200 nM each of ORC, Cdc6 and Mcm2-7-Cdt1 in a total volume of 5 μl of buffer B for 30 min at 30 °C and 1,250 r.p.m. DDK (300 nM) was incubated at equimolar ratio with either wild-type or catalytically dead Rad53 in buffer B supplemented with 5 mM ATP for 15 min at 30 °C and 1,250 r.p.m. As a control, DDK was treated in the same manner but swapping Rad53 for buffer; 5 μl of the DDK/Rad53 mixture was then then added to 5 μl of the loading reaction and incubation was continued for 15 min at 30 °C. Reactions were diluted and EM grids prepared as described above. For analysis of phosphorylation of the MCM DH in this experiment, 2 μl of the reaction was mixed with 2 μl of M280 streptavidin paramagnetic beads (Invitrogen) and 15 μl of buffer A. After 30 min at 30 °C, DNA-bound complexes were washed twice with low-salt buffer and eluted by the addition of micrococcal nuclease (MNase, NEB, 1,000 units) and incubation for 10 min at 37 °C. Eluted complexes were analyzed by SDS–PAGE and silver staining (SilverQuest Silver Stain, Bio-Rad).

### Effect of DDK autophosphorylation on MCM–Cdt1 phosphorylation

DDK (1 μM) was incubated with 5 mM ATP in 4 μl of buffer B for 15 min at 30 °C with mixing at 1,250 r.p.m. The reaction volume was then increased to 8 μl following the addition of Mcm2-7-Cdt1 at 25 nM working concentration. After 15 min the reaction was terminated by the addition of Laemmli sample buffer and heating to 97 °C for 10 min. Samples were analyzed by SDS–PAGE and silver staining.

### Negative-stain EM data collection and image processing

Negative-stain micrographs were collected on a Tecnai G2 Spirit transmission electron microscope operated at 120 keV. Micrographs were acquired at a nominal magnification of ×30,000 (yielding a pixel size of 3.45 Å at the specimen level). All image processing was performed in RELION-3.1 (ref. ^62^), with semiautomated particle picking performed using Topaz v.0.2.5 (ref. ^63^) and CTF correction with Gctf^64^.

### Isolated DDK imaged by cryo-EM

UltrAuFoil R1.2/1.3 300-mesh grids were glow discharged at 40 mA for 5 min using a GloQube glow discharge system (Quorum) and coated with graphene oxide flakes (Sigma). Next, 4 μl of 40 ng μl^–1^ DDK was applied to each grid for 30 s before double-sided blotting for 4 s and plunge-freezing in liquid ethane using a Vitrobot Mark IV (FEI) operated at room temperature with 90% humidity. A total of 9,469 videos were collected at ×165,000 magnification (yielding a pixel size of 0.84 Å at specimen level) on a Titan Krios electron microscope equipped with a K2 Summit direct electron detector (Gatan Inc.).

A total of 2,967,226 particles were picked from motion-corrected video sums using crYOLO^65^. CTF was estimated using Gctf^64^, and 2D classification was performed after extraction of particles in a 240-pixel box in RELION-3.0.7 (ref. ^39^).

### Preparation of MCM DHs in complex with DDK for cryo-EM

DNA (225 ng, 8 nM final concentration) was incubated with ORC and Cdc6 in 45 μl of buffer B, with stirring at 1,250 r.p.m. for 10 min at 30 °C. Mcm2-7-Cdt1 was added for 60 min, resulting in a final concentration of 96 nM for all protein factors. DDK (289 nM) was added for 50 min before plunge-freezing, then 4 μl of the 1:2 diluted reaction (75 ng μl^–1^ total protein concentration) was applied for 30 s to glow-discharged lacey grids (400 mesh) and covered with an ultrathin layer of carbon (Agar Scientific). Grids were double-side blotted for 3 s and plunge-frozen in liquid ethane using a Vitrobot Mark IV (FEI) operated at room temperature with 90% humidity.

### Cryo-EM data collection, image processing and atomic model building

A total of 18,135 videos were collected at ×130,000 magnification (yielding a pixel size of 1.08 Å at the specimen level) on a Titan Krios electron microscope equipped with a K2 Summit direct electron detector and BioQuantum energy filter (Gatan Inc.). Particles were picked from motion-corrected video sums using crYOLO^65^. CTF correction was performed using Gctf^64^. Particles were extracted with a 480-pixel box and subjected to 2D classification in RELION-3.1 (ref. ^62^). MCM DH particles were used for ab initio reconstruction and subsequent homogeneous refinement in cryoSPARC^66^ followed by C2-symmetrized autorefinement in RELION. Particles were polished and subjected to seven rounds of CTF refinement in RELION. After symmetry expansion in RELION, the signal of one of the two DDK molecules was subtracted and the resulting particles were subjected to focused 3D classification without alignment, to identify DDK-engaged MCM DHs. DDK-DH particles were 3D autorefined and postprocessed in RELION. Density modification was performed using ResolveCryoEM^40^ in Phenix^67^. The atomic model of DNA-bound MCM DH was built as follows. The atomic structure of MCM DH, rerefined with Isolde^68^ (PDB entry 6EYC), was refined using Namdinator^45^ against our cryo-EM density and adjusted manually in Coot^46^. DNA was built using Coot. The atomic model for DDK was built in Coot by manual modification of homology models (based on human DDK, PDB entry 6YA7 (ref. ^35^)), which were generated using HHPRED^69^ and I-Tasser^70^. The yeast Dbf4 BRCT domain (PDB entry 3QBZ^34^) was docked into the density using UCSF Chimera^71^. The resulting model was real-space refined in Phenix^47^. Structural figures were prepared using ChimeraX^72^.

### Mass spectrometry analysis of MCM phosphorylation

To assay for loading-competent helicase phosphorylation, 100 nM of Mcm2-7-Cdt1 was incubated with 2 μM wild-type DDK for 30 min at 30 °C, with mixing at 1,250 r.p.m., in 15 μl of buffer A supplemented 5 mM ATP. To assay DH phosphorylation, MCM loading was performed by coincubation of 2.25 nM DNA, 5.5 nM ORC and 5.5 nM Cdc6 in 1 ml of buffer A supplemented with 5 mM ATP. After thermomixer incubation for 10 min at 30 °C, Mcm2-7-Cdt1 was added to a final concentration of 13 nM and incubated for 30 min, followed by the addition of 42 μl of M280 streptavidin paramagnetic beads (Invitrogen) and incubation for 30 min. Wild-type DDK was added to a final concentration of 21 nM and incubated for 30 min. Unbound proteins were removed with three 1-ml washes in high-salt buffer and one in low-salt buffer. DNA-bound complexes were eluted by coincubation of 4,000 units of micrococcal nuclease for 15 min at 37 °C. Each sample was loaded onto a 12% SDS–PAGE gel and separated by running it for 7 mm into the gel. Protein bands excised from the gel were subjected to trypsin digestion. Proteolyzed proteins were analyzed by liquid chromatography–tandem mass spectrometry, adopting the 44-min binary gradient supplied with an Evosep nanoHPLC coupled to an Orbitrap Lumos Tribrid mass spectrometer (Thermo Scientific). Fragmentation was performed by higher-energy collisional dissociation with acquisition in the ion trap using the vendor’s ‘universal’ data-dependent acquisition method. Data were searched using Maxquant (https://www.maxquant.org/) against a recent download of the uniprot *S. cerevisiae* FASTA database, with visualization in Perseus (https://maxquant.net/perseus/).

### Reporting Summary

Further information on research design is available in the Nature Research Reporting Summary linked to this article.

## Online content

Any methods, additional references, Nature Research reporting summaries, source data, extended data, supplementary information, acknowledgements, peer review information; details of author contributions and competing interests; and statements of data and code availability are available at 10.1038/s41594-021-00698-z.

## Supplementary information

Supplementary Information Supplementary Tables 1–4.

Reporting Summary

Peer Review Information

## Data Availability

Cryo-EM maps and atomic model coordinates have been deposited in the Electron Microscopy Data Bank under accession code nos. 13176 (DNA-DH) and 13211 (DNA-DH-DDK), and Protein Data Bank 7P30 (DNA-DH) and 7P5Z (DNA-DH-DDK). Source data are provided with this paper.

## Source data

Source Data Fig. 2 Uncropped SDS–PAGE gel.
Source Data Fig. 3 Uncropped SDS–PAGE gel and autoradiograph.
Source Data Fig. 3 Statistical source data.
Source Data Fig. 4 Uncropped SDS–PAGE gel.
Source Data Fig. 5 Uncropped SDS–PAGE gels and autoradiograph.
Source Data Fig. 6 Uncropped SDS–PAGE gel and autoradiograph.
Source Data Fig. 6 Statistical source data.
Source Data Extended Data Fig. 2 Statistical source data.
Source Data Extended Data Fig. 5 Uncropped SDS–PAGE gels.
Source Data Extended Data Fig. 5 Statistical source data.
