# Supplementary material for: Structural mechanism for the selective phosphorylation of DNA-loaded MCM double hexamers by the Dbf4-dependent kinase
Source: Nat Struct Mol Biol. 2021 Dec 28;29(1):10–20. doi: 10.1038/s41594-021-00698-z (PMC8770131; doi:10.1038/s41594-021-00698-z)
Supplement: Supplementary file 1 — Supplementary Tables 1–4. [file 41594_2021_698_MOESM1_ESM.pdf]

nature portfolio

https://doi.org/10.1038/s41594-021-00698-z

Supplementary information

Structural mechanism for the selective phosphorylation of DNA-loaded MCM double hexamers by the Dbf4-dependent kinase

In the format provided by the authors and unedited

Supplementary Table 1: DDK-dependent phosphorylation of Mcm4 detected by mass spectrometry.

Site	Phosphorylated in Mcm2-7–Cdt1	Localisation probability	PEP	Phosphorylated in MCM double hexamer	Localisation probability	PEP
**S52**	Yes	1.00	0.00036	Yes	1.00	5.87E-29
**S56**	Yes	1.00	0.00036	Yes	1.00	8.31E-20
**S68/S69**	No	-	2.55E-11	Majority unmodified, but some phosphorylation detectable	0.88	1.07E-03
**S75**	No	-	2.55E-11	Majority unmodified, but some phosphorylation detectable	0.99	1.07E-03
**S76**	No	-	2.55E-11	Majority unmodified, but some phosphorylation detectable	0.80	1.07E-03
**S77**	No	-	2.55E-11	Majority unmodified, but some phosphorylation detectable	0.81	1.38E-03
**S82**	No	-	4.57E-06	Yes	1.00	2.84E-05
**S87**	No	-	4.57E-06	Yes	1.00	3.17E-04
**S108/ S112**	Not detected			Only phosphorylated peptide detected		0.044
**S118**	No	-	1.57E-04	Yes	1.00	4.50E-03
**T140**	No	-	1.99E-20	Yes	1.00	2.62E-08
**S141**	No	-	1.99E-20	Yes	1.00	2.62E-08
**S144**	No	-	1.99E-20	Yes	1.00	2.62E-08
**S171**	Only detected in sample without DDK			Not detected		

Supplementary Table 2: DDK-dependent phosphorylation of Mcm6 detected by mass spectrometry.

Site	Phosphorylated in Mcm2-7–Cdt1	Localisation probability	PEP	Phosphorylated in MCM double hexamer	Localisation probability	PEP
**T75**	No	-	3.34E-60	Yes	1.00	1.59E-23
**S78**	No	-	3.34E-60	Yes	1.00	1.59E-23
**S226**	Yes	0.96	4.64E-48	Yes	0.96	S.83E-26
**S232/234**	Yes	0.98	8.07E-06	Yes	0.72	5.32E-10
**S1016/S 1017**	Yes	0.57/0.5	8.16E-14	Yes	0.56/0.5	1.35E-04

Supplementary Table 3: Yeast strains.

Strain	Genotype (all in W303 background)	Reference
yAM33	*MAT**a***<br>*bar1::Hyg*<br>*pep4:: KanMx*<br>*his3::pRS303-Cdt1/Gal4 (HIS3)*<br>*ura3:: ::pRS306-Mcm2/Mcm3 CBP-Mcm3 (URA3)*<br>*trp1::pRS304-Mcm4/5 (TRP1)*<br>*leu2::pRS305-Mcm6/7 (LEU2)*	(Coster et al. 2014)
yJG3	*MAT**a** ade2-1 ura3-1 his3-11,15 trp1-1 leu2-3,112 can1-100,*<br>*pep4::KanMX,*<br>*trp1::TRP1pRS304/CDC7, CBP-DBF4*$^{\Delta 119\text{-}219}$	This study
yJG13	*MAT**a** ade2-1 ura3-1 his3-11,15 trp1-1 leu2-3,112 can1-100,*<br>*pep4::KanMX,*<br>*trp1::TRP1pRS304/CDC7, CBP-DBF4*^{T188D,S192D}	This study
ySDK8	*MAT**a** ade2-1 ura3-1 his3-11,15 trp1-1 leu2-3,112 can1-100,*<br>*pep4::KanMX,*<br>*trp1::TRP1pRS304/CDC7, CBP-DBF4*	(On et al. 2014)
ySD-ORC	*MAT**a** ade2-1 ura3-1 his3-11,15 trp1-1 leu2-3,112 can1-100*<br>*bar1::Hyg*<br>*pep4::KanMX*<br>*his3::pRS303-ORC3/4 (HIS3)*<br>*ura3::pRS306-ORC1/2 CBP-Orc1 (URA3)*<br>*trp1::pRS304-ORC5/6 (TRP1)*	(Frigola et al. 2013)

Supplementary Table 4: DNA plasmids.

Plasmid	Construct	Reference
pAM3	pGEX-6p-1-CDC6	(Frigola et al. 2013)
	pET21b-RAD53	(Gilbert et al. 2001)
pAWM7	pET21b-RAD53^{K227A,D339A}-6xHis	(McClure and Diffley 2021)
pJG1	pRS304-CDC7-GAL-CBP-DBF4$^{\Delta 119\text{-}219}$	This study
pJG9	pRS304-CDC7-GAL-CBP-DBF4^{T188D,S192D}	This study
