# Supplementary material for: Structural mechanism for the selective phosphorylation of DNA-loaded MCM double hexamers by the Dbf4-dependent kinase
Source: Nat Struct Mol Biol. 2021 Dec 28;29(1):10–20. doi: 10.1038/s41594-021-00698-z (PMC8770131; doi:10.1038/s41594-021-00698-z)
Supplement: Supplementary file 2 — Reporting Summary [file 41594_2021_698_MOESM2_ESM.pdf]

nature portfolio

Corresponding author(s): Alessandro Costa

Last updated by author(s): Oct 27, 2021

Reporting Summary

Nature Portfolio wishes to improve the reproducibility of the work that we publish. This form provides structure for consistency and transparency in reporting. For further information on Nature Portfolio policies, see our Editorial Policies and the Editorial Policy Checklist.

Statistics

For all statistical analyses, confirm that the following items are present in the figure legend, table legend, main text, or Methods section.

n/a	Confirmed	
☐	☒	The exact sample size (*n*) for each experimental group/condition, given as a discrete number and unit of measurement
☐	☒	A statement on whether measurements were taken from distinct samples or whether the same sample was measured repeatedly
☒	☐	The statistical test(s) used AND whether they are one- or two-sided<br>*Only common tests should be described solely by name; describe more complex techniques in the Methods section.*
☒	☐	A description of all covariates tested
☒	☐	A description of any assumptions or corrections, such as tests of normality and adjustment for multiple comparisons
☐	☒	A full description of the statistical parameters including central tendency (e.g. means) or other basic estimates (e.g. regression coefficient) AND variation (e.g. standard deviation) or associated estimates of uncertainty (e.g. confidence intervals)
☒	☐	For null hypothesis testing, the test statistic (e.g. *F*, *t*, *r*) with confidence intervals, effect sizes, degrees of freedom and *P* value noted<br>*Give P values as exact values whenever suitable.*
☒	☐	For Bayesian analysis, information on the choice of priors and Markov chain Monte Carlo settings
☒	☐	For hierarchical and complex designs, identification of the appropriate level for tests and full reporting of outcomes
☒	☐	Estimates of effect sizes (e.g. Cohen's *d*, Pearson's *r*), indicating how they were calculated

Our web collection on statistics for biologists contains articles on many of the points above.

Software and code

Policy information about availability of computer code

Data collection	Gatan DigitalMicrograph and ThermoFisher EPU
Data analysis	crYOLO v1.40, Topaz v0.2.5, Gctf v1.06, RELION v3.04 and v3.1, cryoSPARC 2.8, UCSF Chimera v1.14, ChimeraX-1.1.1, COOT v0.9-pre, Phenix v1.19.2, MolProbity, HHPred, I-Tasser, Isolde, Namdinator, Maxquant

For manuscripts utilizing custom algorithms or software that are central to the research but not yet described in published literature, software must be made available to editors and reviewers. We strongly encourage code deposition in a community repository (e.g. GitHub). See the Nature Portfolio guidelines for submitting code & software for further information.

Data

Policy information about availability of data

All manuscripts must include a data availability statement. This statement should provide the following information, where applicable:

- Accession codes, unique identifiers, or web links for publicly available datasets
- A description of any restrictions on data availability
- For clinical datasets or third party data, please ensure that the statement adheres to our policy

The cryo-EM map and atomic model coordinates for the DNA-bound MCM double hexamer are deposited in the Electron Microscopy Data Bank and Protein Data Bank respectively under the accession code 7P30 and EMD-13176. The cryo-EM map and model of the MCM–DDK complex are found under the accession code 7P5Z and EMD-13211.

Field-specific reporting

Please select the one below that is the best fit for your research. If you are not sure, read the appropriate sections before making your selection.

☒ Life sciences ☐ Behavioural & social sciences ☐ Ecological, evolutionary & environmental sciences

For a reference copy of the document with all sections, see nature.com/documents/nr-reporting-summary-flat.pdf

Life sciences study design

All studies must disclose on these points even when the disclosure is negative.

Sample size	In our negative stain EM experiments, we imaged ATP-dependent MCM DH loading and phosphorylation by DDK, yielding multiple reaction intermediates. To determine 2D averages of DHs engaged to DDK, we usually collected 100-250 micrographs and repeated experiments at least 3 times per experimental condition.<br>To obtain high-resolution structures of the DNA-bound DH and DH–DDK complexes from the mixed population of reaction intermediates in the cryo-EM experiment, ~18.1k micrographs were collected from a single grid.<br><br>The number of micrographs collected was decided based on the target resolution of the electron microscopy averages/3D structures.<br><br>No statistical methods were used to predetermine sample size.
Data exclusions	Negative stain and cryo-EM micrographs with poor staining or ice contamination, respectively, were excluded. Picked particles that did not align to a distinct class in 2D and 3D (cryo-EM only) were excluded from further analysis. DHs that were not engaged with DDK were removed from the cryo-EM dataset to yield the best reconstruction of the MCM–DDK complex.
Replication	The MCM–DDK complex identified in this study was visualized in multiple experiments (both negative stain and cryo-EM). Multiple assays (kinase assay, autophosphorylation) confirmed that kinase activity was not compromised in ΔBRCT, while DH engagement and phosphorylation was reproducibly impaired for ΔBRCT (established by negative stain EM and bead-based pull down assays visualized by SDS-PAGE and silver staining). The finding that DDK autophosphorylation inhibits peptide and MCM–Cdt1 phosphorylation, but not MCM double hexamer phosphorylation was confirmed in triplicate experiments. The phosphorylation-dependent inhibition of DDK by Rad53 was confirmed in triplicate kinase assays and duplicate MCM double hexamer phosphorylation visualised by SDS-PAGE and sliver stain and negative stain EM experiments.
Randomization	Randomization of samples is not relevant for a single particle electron microscopy study such as this. Automated particle picking using the same Topaz model and reference free 2D averaging is a solid bias-free approach.
Blinding	Blinding is not relevant for a single particle electron microscopy study such as this. Automated particle picking using the same Topaz model and reference free 2D averaging is a solid bias-free approach.

Reporting for specific materials, systems and methods

We require information from authors about some types of materials, experimental systems and methods used in many studies. Here, indicate whether each material, system or method listed is relevant to your study. If you are not sure if a list item applies to your research, read the appropriate section before selecting a response.

Materials & experimental systems

n/a	Involved in the study
☒	☐ Antibodies
☐	☒ Eukaryotic cell lines
☒	☐ Palaeontology and archaeology
☒	☐ Animals and other organisms
☒	☐ Human research participants
☒	☐ Clinical data
☒	☐ Dual use research of concern

Methods

n/a	Involved in the study
☒	☐ ChIP-seq
☒	☐ Flow cytometry
☒	☐ MRI-based neuroimaging

Eukaryotic cell lines

Policy information about cell lines

Cell line source(s)	S.cerevisiae overexpression strains for DH loading factors and phosphorylation have previously been described in Coster et al 2014, Frigola et al 2013, On et al 2014.
Authentication	S.cerevisiae overexpression strains were checked for correct plasmid integration by PCR amplification from extracted genomic DNA.

Mycoplasma contamination	S.cerevisiae overexpression strains were not tested for mycoplasma contamination.
Commonly misidentified lines (See ICLAC register)	No commonly misidentified cell lines were used in this study.
