# Supplementary figures and images for: Structural mechanism for the selective phosphorylation of DNA-loaded MCM double hexamers by the Dbf4-dependent kinase
Source: Nat Struct Mol Biol. 2021 Dec 28;29(1):10–20. doi: 10.1038/s41594-021-00698-z (PMC8770131; doi:10.1038/s41594-021-00698-z)

Source data

Figure 2a

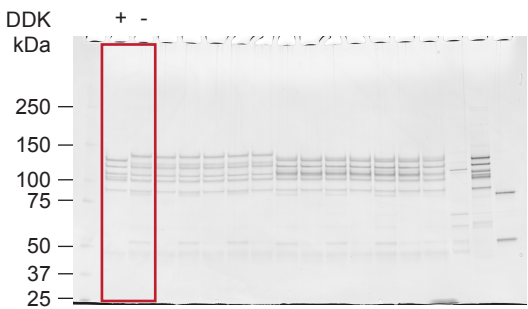

Supplement: Source Data Fig. 2 — Uncropped SDS–PAGE gel. [file 41594_2021_698_MOESM4_ESM.pdf]

Source data

Figure 3c

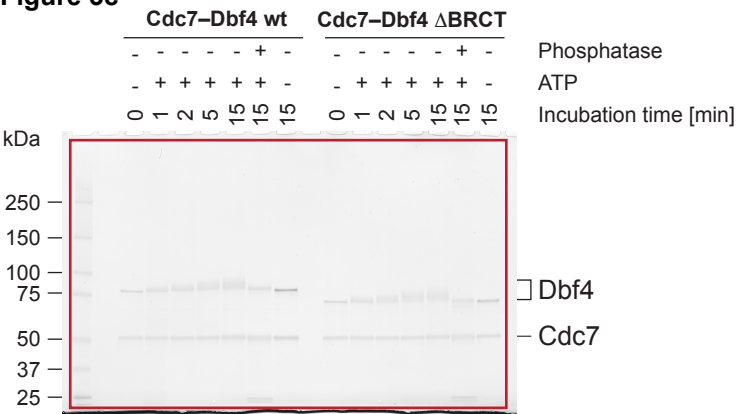

Figure 3d

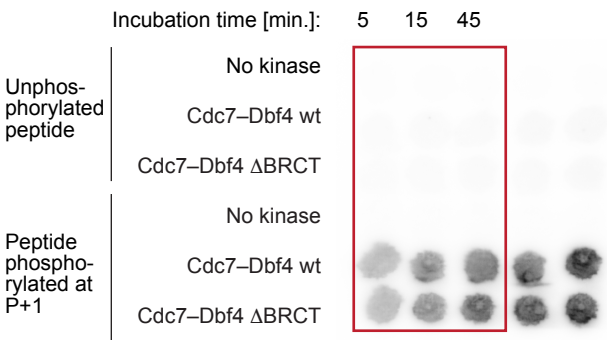

Supplement: Source Data Fig. 3 — Uncropped SDS–PAGE gel and autoradiograph. [file 41594_2021_698_MOESM5_ESM.pdf]

Source data

Figure 4c

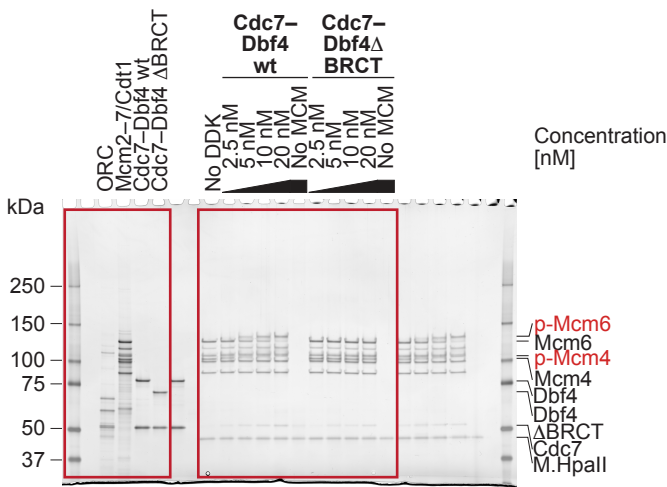

Supplement: Source Data Fig. 4 — Uncropped SDS–PAGE gel. [file 41594_2021_698_MOESM7_ESM.pdf]

Source data

Figure 5a

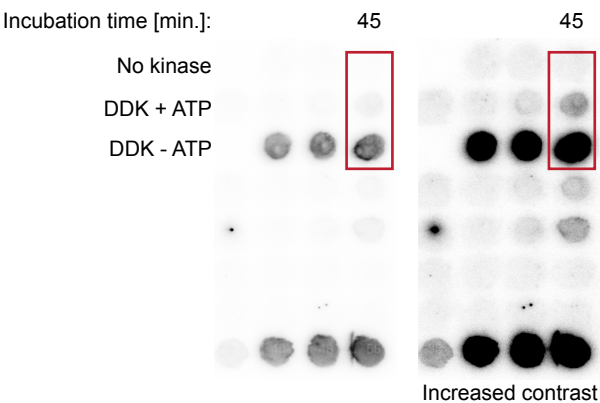

Figure 5b

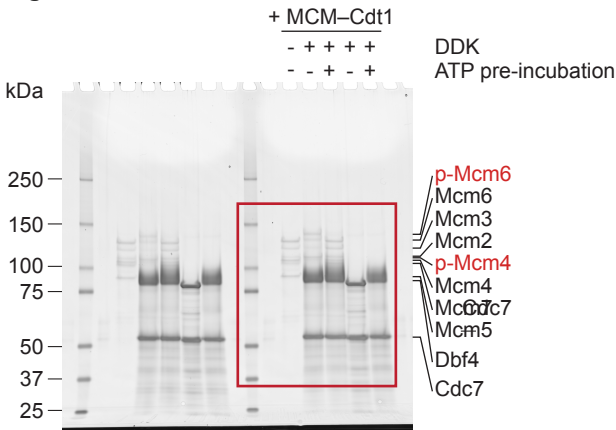

Figure 5c

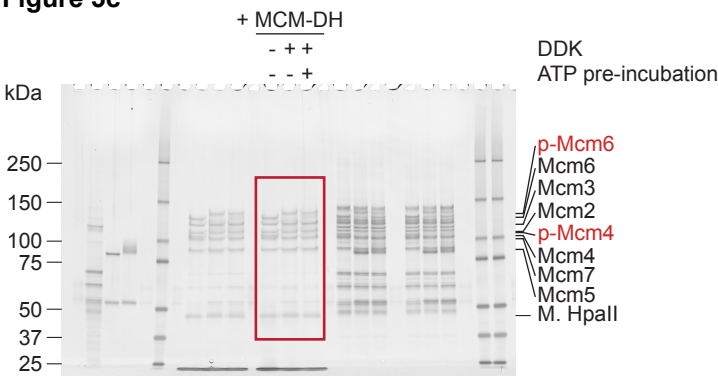

Supplement: Source Data Fig. 5 — Uncropped SDS–PAGE gels and autoradiograph. [file 41594_2021_698_MOESM8_ESM.pdf]

Source data

Figure 6b

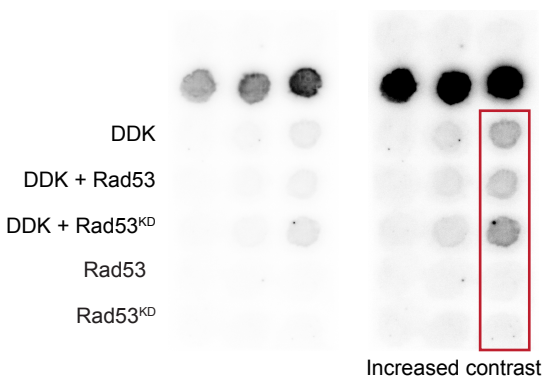

Figure 6d

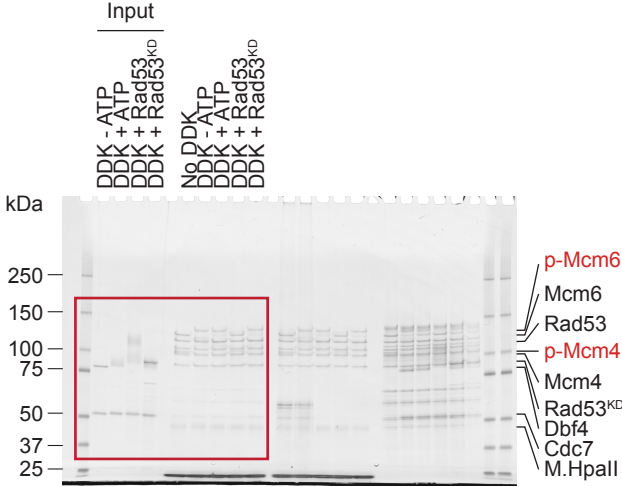

Supplement: Source Data Fig. 6 — Uncropped SDS–PAGE gel and autoradiograph. [file 41594_2021_698_MOESM9_ESM.pdf]

Source data

Extended Data Figure 5a

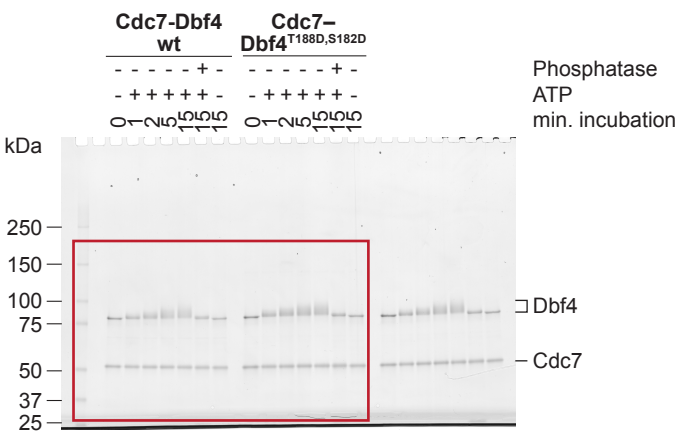

Extended Data Figure 5c

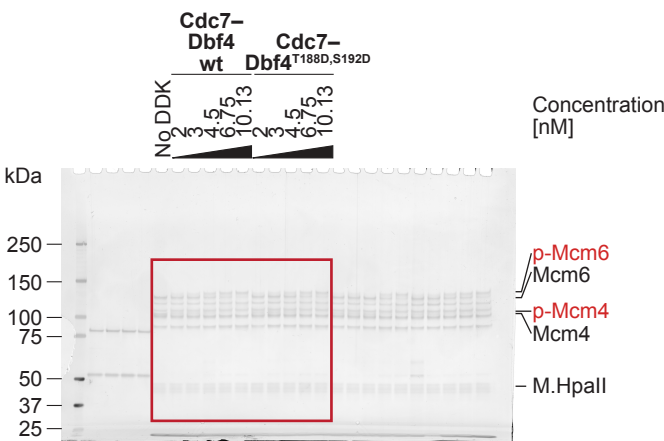

Extended Data Figure 5g

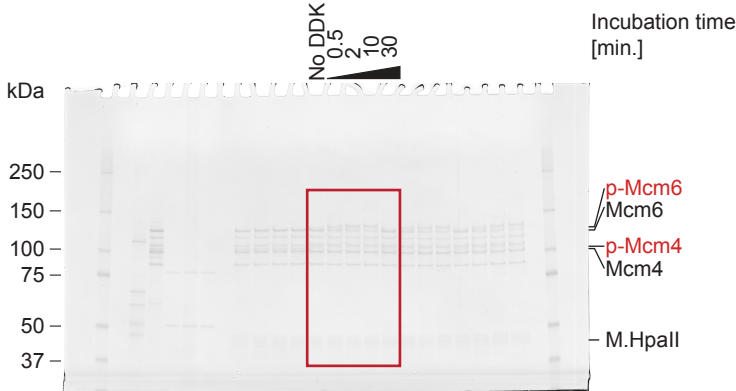

Supplement: Source Data Extended Data Fig. 5 — Uncropped SDS–PAGE gels. [file 41594_2021_698_MOESM12_ESM.pdf]
