# Supplementary material for: Structural mechanism for the selective phosphorylation of DNA-loaded MCM double hexamers by the Dbf4-dependent kinase
Source: Nat Struct Mol Biol. 2021 Dec 28;29(1):10–20. doi: 10.1038/s41594-021-00698-z (PMC8770131; doi:10.1038/s41594-021-00698-z)
Supplement: Supplementary file 3 — Peer Review Information [file 41594_2021_698_MOESM3_ESM.pdf]

Peer Review Information

Journal: Nature Structural and Molecular Biology
Manuscript Title: Structural mechanism for the selective phosphorylation of DNA-loaded MCM double hexamers by the Dbf4-dependent kinase
Corresponding author name(s): Dr Alessandro Costa

Reviewer Comments & Decisions:

Decision Letter, initial version:

27th Sep 2021

Dear Alessandro,

Thank you again for submitting your manuscript "Structural mechanism for the selective phosphorylation of DNA-loaded MCM double hexamers by the Dbf4-dependent kinase". The reports of the referees are copied below, and based on these comments, we are happy to accept your paper, in principle, for publication as an Article in Nature Structural & Molecular Biology, on the condition that you revise your manuscript in response to the comments of the referees and our editorial requirements.

I hope you will be pleased to see that all 3 reviewers are quite positive about the interest and impact of the findings, and that each provides suggestions to clarify or expand on aspects of the study that we agree would strengthen the work and that we would like to see incorporated in a revised manuscript.

The text and figures require revisions. Note that, within a few days, we will send you detailed instructions for the final revision, along with information on editorial and formatting requirements. We recommend that you do not start revising the manuscript until you receive this additional information.

Data availability: this journal strongly supports public availability of data. Please place the data used in your paper into a public data repository, or alternatively, present the data as Supplementary Information. If data can only be shared on request, please explain why in your Data Availability Statement, and also in the correspondence with your editor. Please note that for some data types, deposition in a public repository is mandatory - more information on our data deposition policies and

available repositories can be found below:
https://www.nature.com/nature-research/editorial-policies/reporting-standards#availability-of-data

TRANSPARENT PEER REVIEW
Nature Structural & Molecular Biology offers a transparent peer review option for new original research manuscripts submitted from 1st December 2019. We encourage increased transparency in peer review by publishing the reviewer comments, author rebuttal letters and editorial decision letters if the authors agree. Such peer review material is made available as a supplementary peer review file. Please state in the cover letter ‘I wish to participate in transparent peer review’ if you want to opt in, or ‘I do not wish to participate in transparent peer review’ if you don’t. Failure to state your preference will result in delays in accepting your manuscript for publication.
Please note: we allow redactions to authors’ rebuttal and reviewer comments in the interest of confidentiality. If you are concerned about the release of confidential data, please let us know specifically what information you would like to have removed. Please note that we cannot incorporate redactions for any other reasons. Reviewer names will be published in the peer review files if the reviewer signed the comments to authors, or if reviewers explicitly agree to release their name. For more information, please refer to our FAQ page.

ORCID

Nature Structural & Molecular Biology is committed to improving transparency in authorship. As part of our efforts in this direction, we are now requesting that all authors identified as ‘corresponding author’ create and link their Open Researcher and Contributor Identifier (ORCID) with their account on the Manuscript Tracking System (MTS) prior to acceptance. ORCID helps the scientific community achieve unambiguous attribution of all scholarly contributions. For more information please visit http://www.springernature.com/orcid

For all corresponding authors listed on the manuscript, please follow the instructions in the link below to link your ORCID to your account on our MTS before submitting the final version of the manuscript. If you do not yet have an ORCID you will be able to create one in minutes.
https://www.springernature.com/gp/researchers/orcid/orcid-for-nature-research

IMPORTANT: All authors identified as ‘corresponding author’ on the manuscript must follow these instructions. Non-corresponding authors do not have to link their ORCIDs but are encouraged to do so. Please note that it will not be possible to add/modify ORCIDs at proof. Thus, if they wish to have their ORCID added to the paper they must also follow the above procedure prior to acceptance.

To support ORCID's aims, we only allow a single ORCID identifier to be attached to one account. If you have any issues attaching an ORCID identifier to your MTS account, please contact the Platform Support Helpdesk.

We hope that you will support this initiative and supply the required information. Should you have any query or comments, please do not hesitate to contact me.

In recognition of the time and expertise our reviewers provide to Nature Structural & Molecular Biology's editorial process, we would like to formally acknowledge their contribution to the external peer review of your manuscript entitled "Structural mechanism for the selective phosphorylation of DNA-loaded MCM double hexamers by the Dbf4-dependent kinase". For those reviewers who give their assent, we will be publishing their names alongside the published article.

"Nature Structural & Molecular Biology has now transitioned to a unified Rights Collection system which will allow our Author Services team to quickly and easily collect the rights and permissions required to publish your work. Once your paper is accepted, you will receive an email in approximately 10 business days providing you with a link to complete the grant of rights. If you choose to publish Open Access, our Author Services team will also be in touch at that time regarding any additional information that may be required to arrange payment for your article.

Please note that you will not receive your proofs until the publishing agreement has been received through our system.

For information regarding our different publishing models please see our Transformative Journals page. If you have any questions about costs, Open Access requirements, or our legal forms, please contact ASJournals@springernature.com.."

If you have any questions, please do not hesitate to contact me directly.

With kind regards,

Beth

Beth Moorefield, Ph.D.
Senior Editor
Nature Structural & Molecular Biology

Reviewer #1 [expertise: structural biology/DNA replication]
(Remarks to the Author):

The manuscript by Miller et al investigates an important question in the field of DNA replication, namely how the MCM double hexamer is specifically recognized and phosphorylated by DDK. Their structures are well done, address many open questions, and raise new questions. The trans-phosphorylation mechanism illustrated here is very cool and intuitive. I support publication with minor revisions.

Minor comments:
1) The authors describe the structure of the DNA-DH-DDK ternary complex, but it is hard to tell in their figures how strong the density for DDK actually is. More clear representation of this density is necessary to fully assess the level of uncertainty in their placement of the DDK proteins.
2) In lines 199 through 201, the authors state: "Given the multiple phosphorylation targets in the amino-terminal Mcm4, it is not surprising that visible cryo-EM density for the Mcm4 phosphorylation substrate is lacking within the active site." Just a clarification: is there no visible density at all in the active site or is there unfittable density? Furthermore, what is the phosphorylation status of this complex? Did the authors determine the structure of a post-phosphoryl transfer complex? If so, that could explain why there is no density in the active site.
3) The authors don't describe how Mcm6 can get phosphorylated by DDK. How far is Mcm6 and is it a reasonable distance for phosphorylation sites to occur from DDK? I recognize that Mcm6 phosphorylation is not as important as Mcm4 phosphorylation, but I would like a little more explanation as to what the authors think is going on with Mcm6.
4) The authors report the localization of the Dbf4 BRCT domain but the figures make it hard to assess the quality of the fit. Because the maps look a bit sparse in this region, I think it worthwhile to show the reader a closer view so that they can better assess whether the fitting is accurate or not. This could be placed in supplement.

Reviewer #2 [expertise: DNA replication mechanisms]
(Remarks to the Author):

The manuscript by Costa and colleagues describes the structural analysis of Dbf4-dependent kinase (DDK) recognition of the loaded Mcm2-7 double hexamer (McmDH). Using cryo-electron microscopy, the authors determine the structure of the DDK bound to the McmDH. They find that DDK interacts with subunits on both of the Mcm complexes in the double hexamer providing a clear mechanism to explain the previous observation that DDK preferentially modifies Mcm2-7 in this context. Using biochemical observations they provide evidence that the structure observed is required for specific recognition of

the McmDH by DDK. Interestingly, they also find data that suggests that autophosphorylation by DDK enhances specificity of DDK for the McmDH. Finally, they look into the mechanism of Rad53 inhibition of DDK activity, providing evidence that Rad53 phosphorylation of DDK prevents DDK association with the McmDH.

Overall, this manuscript provides strong evidence for a bipartite interaction between DDK and the McmDH that spans the two Mcm2-7 molecules that are assembled at a licensed origin. In addition, the studies provide new insights into the mechanism of Rad53 inhibition of DDK function, the highlight being the inhibition of DDK-McmDH complex formation. There are minor changes that could improve the manuscript (see below), however, this data and the conclusions that are based upon it are important and will be of interest to both the DNA replication and cell cycle fields, as well as those interested in the mechanisms controlling kinase function.

Specific points:

1. The first part of the results discussing the DNA interactions is poorly described. In addition to the single example in the results regarding differences in the DH and the CMG DNA interactions, they should include something in the associated supp figure (Extended data figure 3) to indicate the interactions that are the same of different. This could be handled similarly to the hypothesis that Rad53 sterically inhibits DDK function in extended data figure 5, for example. Alternatively, this could be left out as it is relatively off-topic from the focus of the paper.

2. Although it is true that phosphorylation of Mcm4 is sufficient to drive origin activation, mutation of Mcm4 and Mcm6 phosphorylation sites together are required for observable growth phenotypes. This leads to the conclusion that phosphorylation of either tail is sufficient which should be mentioned. Based on the structure, one might imagine that phosphorylation of Mcm4 is preferred or faster. Do the authors have any information about either of these possibilities (e.g. time courses showing Mcm4 phosphorylation before Mcm6)?

3. Along similar lines, it would be good for the authors to address how they think that the Mcm6 tail is phosphorylated. Do they think that the interaction with Mcm4 NTE (is maintained and the Mcm6 NTE simply binds the Cdc7 active site. Or do they believe that DDK must release from Mcm4 to phosphorylate Mcm6. Is there evidence of DDK tethered just at Mcm2 by the Dbf4-BRCT motif? Do the authors expect mutations that altering the residues of Cdc7 interacting with the Mcm4 NTE residues (177-155) would specifically eliminate Mcm4 modification or modification of Mcm6 and Mcm4?

4. The authors report that only 10% of the original particles are used for analysis (Extended data figure

4). Some description of what the the other particles represent should be included. Are they all intermediates prior to DH formation?

Reviewer #3 [DNA replication/repair]

(Remarks to the Author):

Review of "Structural mechanism for the selective phosphorylation of DNA-loaded MCM double hexamers by the Dbf4-dependent kinase" by Greiwe et al for NSMB. This manuscript describes the cryo-EM structure of the loaded MCM double hexamer in complex with DDK and provides evidence for an in trans phosphorylation mechanism to activate opposing hexamers for initiation of replication. They elegantly detail the BRCT domain interacting with MCM2 and the C-terminal DBf4/Cdc7 phosphorylating MCM4 on the other hexamer. They validate their results with deletion constructs of Dbf4 that abrogate phosphorylation, in vitro peptide phosphorylation assays that highlight a dual mechanism for inhibition by Rad53 explain its role in halting late origin firing during DDR, and mass spectrometry to map phosphosites. However most impressive is the overall characterization of the structural function relationship of MCM double hexmaer -- DDK trans activation -- Rad53 inhibition of DDK/Cdc7. This work is very high quality, the manuscript is well written and organized, and the overall results are conclusions are extremely important to the field. I have very few comments, except to say really nice work.

1) the in vitro phosphorylation assay in Figure 5b is nicely quantified with three replicates. Is there a significant difference between the first bar (DDK alone) and the third bar (KD)? What is the reason for increased phosphorylation when Rad53 is inactive?
2) Do you have any density for a Dbf4-MCM6 interaction which also gets phosphorylated. Is it that the linker just allows the Dbf4/Cdc7 domain to swing over to MCM6 as well? Would this interaction withe MCM6 have less affinity?
3) Can you quantify the amount (or %) of MCM4 and MCM6 that gets phosphorylated? looks like in your gels (Figs 4h, 5d) that there is almost complete phosphorylation of MCM6 and only partial phosphorylation of MCM4, yet your focus in this paper is on MCM4. How can you explain the result that MCM6 is phosphorylated more, yet the more stable structure is with MCM4?
4) Did you also characterize MCM6 phosphosites by MS? Why are they not included? Was there anything new there?
5) Fig 5d, why does there appear to be phosphorylation of MCM6 (and maybe MCM4) in lane 6 when ATP is absent?
6) End of discussion, the Champasa 2019 reference is missing in the Bibliography

Author Rebuttal to Initial comments

We would like to thank the reviewers for the positive assessment of our work. A point-by-point response to the issues raised is found below.

Reviewer #1

We are pleased to learn that the reviewer considers the question investigated “important”, the structural work “well done”, the mechanism “very cool and intuitive” and that the reviewer “support(s) publication with minor revisions”.

Minor comments:

1) The authors describe the structure of the DNA-DH-DDK ternary complex, but it is hard to tell in their figures how strong the density for DDK actually is. More clear representation of this density is necessary to fully assess the level of uncertainty in their placement of the DDK proteins.

Thank you for raising an important point. A new panel has introduced new cut-through views in Figure 2c to better represent how our atomic model matches the cryo-EM density.

2) In lines 199 through 201, the authors state: “Given the multiple phosphorylation targets in the amino-terminal Mcm4, it is not surprising that visible cryo-EM density for the Mcm4 phosphorylation substrate is lacking within the active site.” Just a clarification: is there no visible density at all in the active site or is there unfittable density?

No density could be visualised. We modified the main text to clarify this point.

Furthermore, what is the phosphorylation status of this complex? Did the authors determine the structure of a post-phosphoryl transfer complex? If so, that could explain why there is no density in the active site.

This is a useful remark. Given our mass-spectrometry analysis indicating that most though not all phosphorylations important for activation are dectected in our sample, we believe that the structure might represent a mixture of double hexamers caught in the act of being phosphorylated and of post-phosphoryl transfer complex. We have modified the main text to include this suggestion.

3) The authors don't describe how Mcm6 can get phosphorylated by DDK. How far is Mcm6 and is it a reasonable distance for phosphorylation sites to occur from DDK? I recognize that Mcm6 phosphorylation is not as important as Mcm4 phosphorylation, but I would like a little more explanation as to what the authors think is going on with Mcm6.

We have modified the text and now entertain the possibility that the N-terminal tail of Mcm6, which serves as a substrate for phosphorylation, might reach the Cdc7 active site when DDK is bound to Mcm4. Alternatively, we suggest the possibility that DDK might remain docked onto Mcm2 but engage Mcm6, though we failed to observe such structural transition, at least in the experimental conditions employed.

4) The authors report the localization of the Dbf4 BRCT domain but the figures make it hard to assess the quality of the fit. Because the maps look a bit sparse in this region, I think it worthwhile to show the reader a closer view so that they can better assess whether the fitting is accurate or not. This could be placed in supplement.

We have improved Figure 3b to better depict the quality of the BRCT docking solution.

Reviewer #2

We are pleased to learn that the reviewer thinks that the evidence provided in support of our model is "strong", that the conclusions are "important" and the study will be "of interest" to researchers in DNA replication, cell cycle and kinase mechanisms.

1. The first part of the results discussing the DNA interactions is poorly described. In addition to the single example in the results regarding differences in the DH and the CMG DNA interactions, they should include something in the associated supp figure (Extended data figure 3) to indicate the interactions that are the same of different. This could be handled similarly to the hypothesis that Rad53 sterically inhibits DDK function in extended data figure 5, for example. Alternatively, this could be left out as it is relatively off-topic from the focus of the paper.

We thank the reviewer for an important remark. As comparisons between phosphorylated DHs and CMG are indeed off-topic, we decided to follow their suggestion and remove any mention of CMG (and F363) from the main text.

2. Although it is true that phosphorylation of Mcm4 is sufficient to drive origin activation, mutation of

Mcm4 and Mcm6 phosphorylation sites together are required for observable growth phenotypes. This leads to the conclusion that phosphorylation of either tail is sufficient which should be mentioned.

We now mention this finding in the introduction section.

Based on the structure, one might imagine that phosphorylation of Mcm4 is preferred or faster. Do the authors have any information about either of these possibilities (e.g. time courses showing Mcm4 phosphorylation before Mcm6)?

This is a great point and we do have titration and time-course data that supports the statement that DDK engagement of N-terminal Mcm4 results in preferred/faster phosphorylation of this subunit. The titration is shown in Figure 4c (as in the original submission) and a time-course gel is now included in Extended Data Figure 5.

3. Along similar lines, it would be good for the authors to address how they think that the Mcm6 tail is phosphorylated. Do they think that the interaction with Mcm4 NTE (is maintained and the Mcm6 NTE simply binds the Cdc7 active site. Or do they believe that DDK must release from Mcm4 to phosphorylate Mcm6. Is there evidence of DDK tethered just at Mcm2 by the Dbf4-BRCT motif? Do the authors expect mutations that altering the residues of Cdc7 interacting with the Mcm4 NTE residues (177-155) would specifically eliminate Mcm4 modification or modification of Mcm6 and Mcm4?

As mentioned in the reply to Reviewer 1 Point 3, we have modified the text to entertain two possibilities: either Mcm6 phosphorylation happens while the DDK catalytic core binds Mcm4 or DDK swaps from interacting with Mcm4 to Mcm6. The structural data we obtain however does not support either models. In fact, under our experimental conditions, we obtained no evidence of DDK docking to Mcm2 only, which would support flexible substrate engagement by the kinase core. Given that we don’t know how the dispensable Mcm6 substrate becomes phosphorylated, we cannot comment on whether changing Mcm4-interacting residues on Cdc7 would selectively abrogate Mcm4 and not Mcm6 modification.

4. The authors report that only 10% of the original particles are used for analysis (Extended data figure 4). Some description of what the the other particles represent should be included. Are they all intermediates prior to DH formation?

We modified Extended Data Figure 4 to state that 13% of picked particles were loading intermediates (already described in Extended Data Figure 1) and the rest were ‘junk’ particles.

Reviewer #3

We thank this reviewer for deeming our work “elegant”, “very high quality” and “really nice”, our DH/DDK/Rad53 structure/function characterisation “most impressive”, our manuscript “well written and organized”, and our conclusions “extremely important to the field”.

1) the in vitro phosphorylation assay in Figure 5b is nicely quantified with three replicates. Is there a significant difference between the first bar (DDK alone) and the third bar (KD)? What is the reason for increased phosphorylation when Rad53 is inactive?

We believe that the increased MCM peptide phosphorylation plotted in the bar chart reported in Figure 5b is not significant, given the larger error bar associated with the kinase dead Rad53 experiment. In support of this statement, kinase dead Rad53 does not stimulate DH phosphorylation (as shown in 5d) and does not affect origin dependent DNA replication as shown in recently published *in vitro* reconstitution experiments (McClure et al Elife 2021).

2) Do you have any density for a Dbf4-MCM6 interaction which also gets phosphorylated. Is it that the linker just allows the Dbf4/Cdc7 domain to swing over to MCM6 as well?

This is an excellent question, also raised by the other reviewers. Please refer to our replies to Reviewer 1 point 3 and Reviewer 2 point 3.

Would this interaction withe MCM6 have less affinity?

Based on our data, we are not in the position of commenting on the DDK affinity for Mcm6, as we cannot visualise such interaction.

3) Can you quantify the amount (or %) of MCM4 and MCM6 that gets phosphorylated? looks like in your gels (Figs 4h, 5d) that there is almost complete phosphorylation of MCM6 and only partial phosphorylation of MCM4, yet your focus in this paper is on MCM4. How can you explain the result that MCM6 is phosphorylated more, yet the more stable structure is with MCM4?

We realise that the Mcm4 and Mcm6 shifts in the protein gels are difficult to follow when as many as 9 factors are labelled. In Figures 4h and 5d, Mcm4 is fully phosphorylated and Mcm6 only partially phosphorylated. Not the opposite. This is what one would expect given our structure of Mcm4 engaged

by the DDK kinase core. We now improved the labelling of our figure by following the colour code of the cryo-EM structure. In doing so we changed the red labels to Mcm4 (which is now green) and Mcm6 (which is now orange). The point raised helped us generate a better figure and highlight a subtle, yet key, difference. Thank you.

4) Did you also characterize MCM6 phosphosites by MS? Why are they not included? Was there anything new there?

We find that, as previously reported, in the DH Thr75 is selectively phosphorylated by DDK. The same is true for Ser78. Certain DDK depedent phosphorylations can instead be observed both for the DH as well as MCM-Cdt1. These include Ser226, Ser232 (or Ser234) and Ser1016 (or Ser1017). We modified the text to refer to these modifications, which are now reported in the new Supplementary Table 3.

5) Fig 5d, why does there appear to be phosphorylation of MCM6 (and maybe MCM4) in lane 6 when ATP is absent?

The reviewer is perfectly right. The labelling in Figure 5d were confusing. "-ATP" indicates lack of ATP pre-incubation, which is a Rad53 control. ATP is indeed included in the DH phosphorylation reaction and this is why Mcm4 and Mcm6 are phosphorylated. We modified the figure labelling to clarify this point.

6) End of discussion, the Champasa 2019 reference is missing in the Bibliography

Thank you for spotting this. Fixed.

Final Decision Letter:

5th Nov 2021

Dear Alessandro,

We are now happy to accept your revised paper "Structural mechanism for the selective phosphorylation of DNA-loaded MCM double hexamers by the Dbf4-dependent kinase" for publication as a Article in Nature Structural & Molecular Biology.

Acceptance is conditional on the manuscript's not being published elsewhere and on there being no announcement of this work to the newspapers, magazines, radio or television until the publication date in Nature Structural & Molecular Biology.

Before the manuscript is sent to the printers, we shall make any detailed changes in the text that may be

necessary either to make it conform with house style or to make it intelligible to a wider readership. If the changes are extensive, we will ask for your approval before the manuscript is laid out for production. Once your manuscript is typeset you will receive a link to your electronic proof via email within 20 working days, with a request to make any corrections within 48 hours. Please read proofs with great care to make sure that the sense has not been altered. If you have queries at any point during the production process then please contact the production team at rjsproduction@springernature.com. Once your paper has been scheduled for online publication, the Nature press office will be in touch to confirm the details.

Please note that due to tight production schedules, proofs should be returned as quickly as possible to avoid delaying publication. If you anticipate any limitations to your availability over the next 2-4 weeks (such as vacation or traveling to conferences, etc.), please e-mail rjsproduction@springernature.com as soon as possible. Please provide specific dates that you will be unavailable and provide detailed contact information for an alternate corresponding author if necessary.

To assist our authors in disseminating their research to the broader community, our SharedIt initiative provides all co-authors with the ability to generate a unique shareable link that will allow anyone (with or without a subscription) to read the published article. Recipients of the link with a subscription will also be able to download and print the PDF.

As soon as your article is published, you can generate your shareable link by entering the DOI of your article here: <a href="http://authors.springernature.com/share">http://authors.springernature.com/share<a>. Corresponding authors will also receive an automated email with the shareable link

Note the policy of the journal on data deposition: http://www.nature.com/authors/policies/availability.html.

Your paper will be published online soon after we receive proof corrections and will appear in print in the next available issue. You can find out your date of online publication by contacting the production team shortly after sending your proof corrections. Content is published online weekly on Mondays and Thursdays, and the embargo is set at 16:00 London time (GMT)/11:00 am US Eastern time (EST) on the day of publication. Now is the time to inform your Public Relations or Press Office about your paper, as they might be interested in promoting its publication. This will allow them time to prepare an accurate and satisfactory press release. Include your manuscript tracking number (NSMB-A45260A) and our journal name, which they will need when they contact our press office.

About one week before your paper is published online, we shall be distributing a press release to news

organizations worldwide, which may very well include details of your work. We are happy for your institution or funding agency to prepare its own press release, but it must mention the embargo date and Nature Structural & Molecular Biology. If you or your Press Office have any enquiries in the meantime, please contact press@nature.com.

You can now use a single sign-on for all your accounts, view the status of all your manuscript submissions and reviews, access usage statistics for your published articles and download a record of your refereeing activity for the Nature journals.

If you have not already done so, we strongly recommend that you upload the step-by-step protocols used in this manuscript to the Protocol Exchange. Protocol Exchange is an open online resource that allows researchers to share their detailed experimental know-how. All uploaded protocols are made freely available, assigned DOIs for ease of citation and fully searchable through nature.com. Protocols can be linked to any publications in which they are used and will be linked to from your article. You can also establish a dedicated page to collect all your lab Protocols. By uploading your Protocols to Protocol Exchange, you are enabling researchers to more readily reproduce or adapt the methodology you use, as well as increasing the visibility of your protocols and papers. Upload your Protocols at www.nature.com/protocolexchange/. Further information can be found at www.nature.com/protocolexchange/about.

An online order form for reprints of your paper is available at https://www.nature.com/reprints/author-reprints.html. Please let your coauthors and your institutions' public affairs office know that they are also welcome to order reprints by this method.

Please note that <i>Nature Structural & Molecular Biology</i> is a Transformative Journal (TJ). Authors may publish their research with us through the traditional subscription access route or make their paper immediately open access through payment of an article-processing charge (APC). Authors will not be required to make a final decision about access to their article until it has been accepted. Find out more about Transformative Journals

<B>Authors may need to take specific actions to achieve compliance with funder and institutional open access mandates. For submissions from January 2021, if your research is supported by a funder that requires immediate open access (e.g. according to Plan S principles) then you should select the gold OA route, and we will direct you to the compliant route where possible.

For authors selecting the subscription publication route our standard licensing terms will need to be accepted, including our self-archiving policies. Those standard licensing terms will supersede any other terms that the author or any third party may assert apply to any version of the manuscript.

In approximately 10 business days you will receive an email with a link to choose the appropriate publishing options for your paper and our Author Services team will be in touch regarding any additional information that may be required.

You will not receive your proofs until the publishing agreement has been received through our system.

If you have any questions about our publishing options, costs, Open Access requirements, or our legal forms, please contact ASJournals@springernature.com

Sincerely,

Beth

Beth Moorefield, Ph.D.
Senior Editor
Nature Structural & Molecular Biology
